# Effects of error, chimera, bias, and GC content on the accuracy of amplicon sequencing

**DOI:** 10.1128/msystems.01025-23

**Published:** 2023-12-01

**Authors:** Yujia Qin, Liyou Wu, Qiuting Zhang, Chongqin Wen, Joy D. Van Nostrand, Daliang Ning, Lutgarde Raskin, Ameet Pinto, Jizhong Zhou

**Affiliations:** 1 Department of Microbiology and Plant Biology, Institute for Environmental Genomics, University of Oklahoma, Norman, Oklahoma, USA; 2 Fisheries College, Guangdong Ocean University, Zhanjiang, Guangdong, China; 3 Department of Civil and Environmental Engineering, University of Michigan, Ann Arbor, Michigan, USA; 4 School of Civil and Environmental Engineering, Georgia Institute of Technology, Atlanta, Georgia, USA; 5 State Key Joint Laboratory of Environment Simulation and Pollution Control, School of Environment, Tsinghua University, Beijing, China; 6 Earth Sciences Division, Lawrence Berkeley National Laboratory, Berkeley, California, USA; 7 School of Civil Engineering and Environmental Sciences, University of Oklahoma, Norman, Oklahoma, USA; 8 School of Computer Science, University of Oklahoma, Norman, Oklahoma, USA; University of California San Diego, La Jolla, California, USA

**Keywords:** targeted gene amplicon sequencing, MiSeq, chimeric sequence, sequence error, sequence GC content, bias

## Abstract

Targeted amplicon sequencing is widely used in microbial ecology studies. However, sequencing artifacts and amplification biases are of great concern. To identify sources of these artifacts, a systematic analysis was performed using mock communities comprised of 16S rRNA genes from 33 bacterial strains. Our results indicated that while sequencing errors were generally isolated to low-abundance operational taxonomic units, chimeric sequences were a major source of artifacts. Singleton and doubleton sequences were primarily chimeras. Formation of chimeric sequences was significantly correlated with the GC content of the targeted sequences. Low-GC-content mock community members exhibited lower rates of chimeric sequence formation. GC content also had a large impact on sequence recovery. The quantitative capacity was notably limited, with substantial recovery variations and weak correlation between anticipated and observed strain abundances. The mock community strains with higher GC content had higher recovery rates than strains with lower GC content. Amplification bias was also observed due to the differences in primer affinity. A two-step PCR strategy reduced the number of chimeric sequences by half. In addition, comparative analyses based on the mock communities showed that several widely used sequence processing pipelines/methods, including DADA2, Deblur, UCLUST, UNOISE, and UPARSE, had different advantages and disadvantages in artifact removal and rare species detection. These results are important for improving sequencing quality and reliability and developing new algorithms to process targeted amplicon sequences.

**IMPORTANCE:**

Amplicon sequencing of targeted genes is the predominant approach to estimate the membership and structure of microbial communities. However, accurate reconstruction of community composition is difficult due to sequencing errors, and other methodological biases and effective approaches to overcome these challenges are essential. Using a mock community of 33 phylogenetically diverse strains, this study evaluated the effect of GC content on sequencing results and tested different approaches to improve overall sequencing accuracy while characterizing the pros and cons of popular amplicon sequence data processing approaches. The sequencing results from this study can serve as a benchmarking data set for future algorithmic improvements. Furthermore, the new insights on sequencing error, chimera formation, and GC bias from this study will help enhance the quality of amplicon sequencing studies and support the development of new data analysis approaches.

## INTRODUCTION

Amplicon sequencing of targeted genes is a ubiquitous tool in microbial ecology research owing to advances in high-throughput sequencing technologies (1–8), particularly the Illumina MiSeq system (1, 4, 7, 9–12). These high-throughput technologies have enabled the rapid acquisition of microbial community structure and composition information, allowing scientists to readily analyze microbial communities and address interesting hypotheses in microbial biodiversity and biogeography (13). Frequently targeted genes include both phylogenetic markers, like 16S rRNA genes (14–20), internal transcribed spacer, 28S rRNA genes (21–26), and 18S rRNA genes (16, 27–30), and functional genes, such as *nif*H (16, 31–35), *nir*K and *nir*S (36, 37), *nos*Z (36, 38), and *dsr*A and *dsr*B (39, 40).

High-throughput amplicon sequencing revealed the “rare biosphere,” the enormous number of low-abundance taxa present in microbial communities (41–45). This population is important as seed banks or gene pools, which help maintain functional redundancy and robustness within an ecosystem (46, 47). Identifying and mapping the distribution of rare species represent a crucial prerequisite to understanding the biodiversity patterns and trends of these species (48). However, even with high-throughput sequencing technologies, detection of rare species represents a major technical challenge. Both type I (incorrectly reject true rare species) and type II (misclassification of artifacts as species) errors can be generated during library preparation, sequencing, and data processing (48). Even after quality trimming to remove noise and error-prone sequences (49), low-abundance operational taxonomic units (OTUs), such as singletons, doubletons, and tripletons, usually account for a large proportion of the remaining OTUs (48). Studies using 454 pyrosequencing suggest that most of these low-abundance OTUs contain multiple errors or are themselves artifacts such as chimeras (50). However, they may also represent true rare species (51–53). Therefore, there arises a problem in that data filtering may simultaneously remove true rare species while failing to completely remove artifacts.

Amplification bias, artifacts, and errors introduced during library generation, sequencing, and data processing affect the estimation of various diversity indices used to assess microbial communities. Variation introduced by random sampling and low sampling efforts could lead to an overestimation of microbial community β-diversity (10, 54–60). Chao (61) estimates rely heavily on the number of species observed only once to extrapolate the total number of species in a community; thus, it is highly sensitive to the presence of erroneous sequences and artifacts (48). Due to these challenges, great caution must be taken in interpreting high-throughput amplicon sequencing data (8). As such, appropriate sample preparation methods and analysis pipelines should be selected based on the objectives of a study, especially when dealing with low-abundance sequence reads (48).

Illumina sequencing errors have been characterized for both 16S rRNA gene amplicons and metagenomes (62, 63). Most of the observed errors were substitution type miscalls (11) resulting primarily from cross-talk between the emission spectra of the different fluorophores (A/C or G/T) (63) or from phasing or pre-phasing when sequences are synthesized (64). Another source of error noted was from specific sequence regions, such as inverted repeats or GGG sequences (65). All of these types of errors would likely be reflected in the corresponding quality scores. Incorporating phasing primers with varying spacer lengths (0–7 bases) in PCR library preparation can significantly enhance the base diversity of sequencing libraries and improve the quality of sequence data in MiSeq runs (7). However, other types of errors and artifacts, specifically those originating from sample preparation steps, would not correlate with the quality score (63).

In this study, we used mock communities containing near full-length 16S rRNA gene sequences from 33 bacterial strains, representing 27 different phyla, to identify sources of error, artifacts, and biases in MiSeq amplicon sequencing. We compared error rates from different methods of PCR library preparation. Our results indicated that chimeric sequences were the major source of sequencing artifacts, with some artifacts resulting from contamination, while sequence errors were generally restricted to low-abundance OTUs. In addition, GC content of a target gene sequence significantly impacted chimeric sequence formation, strain detection, and sequence quality. Using a two-step PCR method greatly reduced the number of chimeric sequences. These results are important in sequence processing and data interpretation.

## RESULTS

### Data statistics

Three bacterial mock communities (Bm1, Bm2, and Bm3) were amplified using three methods: non-phasing, one-step phasing, and two-step phasing (7). In contrast to the non-phasing and one-step phasing methods, which included a single 30-cycle PCR, the two-step phasing method included an initial 10-cycle PCR with template-specific primers, followed by a 20-cycle PCR with phasing primers. Each mock community was replicated 24 times, with a unique barcode for each replicate, resulting in a total of 216 libraries (3 mock communities × 3 amplification methods × 24 replicates). After sequencing, each mock community library had between 5,688 and 36,012 raw reads (Table S1). Sequences of all libraries were rarefied at 5,688 for amplicon sequence variant (ASV)/OTU classification; other analyses used all reads.

### Chimeric sequences

**Fig 1:**
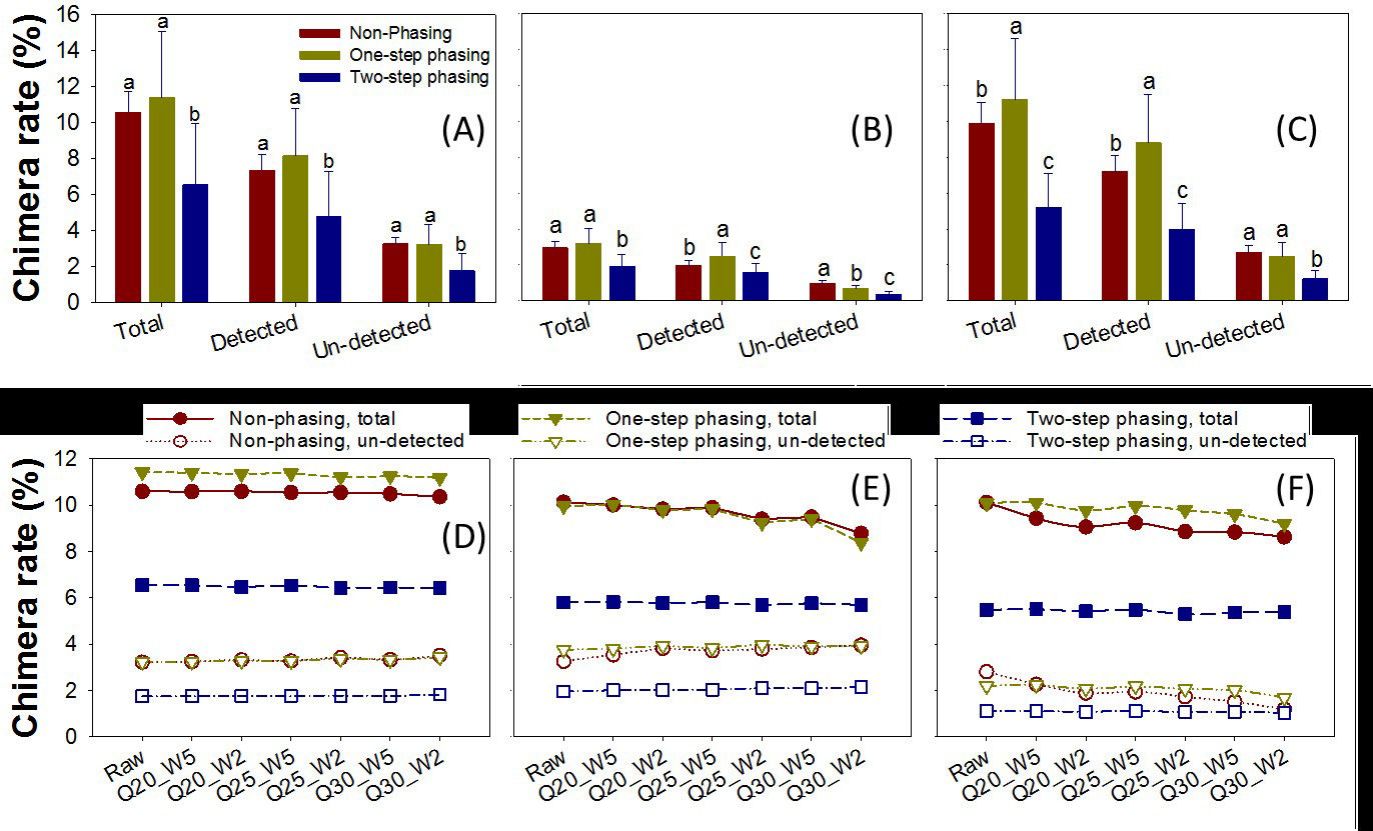
Chimera rate. (**A, B, and C**) Chimera rates for joined raw sequences of Bm1, Bm2, and Bm3. Total chimeras were identified by UCHIME with the mock community 16S rRNA gene sequences as the references. Detected chimeras were those detected by UCHIME (usearch v5.2.3) with Greengenes core database for 16S rRNA gene sequences as the references. The un-detected chimeras were those not detected by UCHIME with Greengenes core database, which remained after chimera removing process. Chimera rate in the three categories is the percentage of chimeric sequences out of all sequences. The average chimera rate for non-phasing libraries (dark red), one-step phasing libraries (dark yellow), and two-step phasing (blue) are shown. Error bars indicate standard deviation of replicate samples. Lowercase letters (i.e., a, b, and c) above the error bars show the results of analysis of variance (ANOVA) and least significant difference (LSD) tests to examine the significant differences, where groups labeled with different lowercase letter are significantly different (*P* < 0.05), while those labeled with the same lowercase letter are not significantly different (*P* > 0.05). Rates of total chimeras and un-detected chimeras were calculated for joined (**D**), forward (**E**), and reverse (**F**) sequences before and after quality trimming at stringencies from Q20-W5 through Q30-W2 of Bm1. The average total chimera rate (long dash line) for non-phasing libraries (dark red circle), one-step phasing (dark yellow triangle), and two-step phasing (blue square) libraries and un-detected chimera rate (dotted line) for non-phasing libraries (dark red open circle), one-step phasing (dark yellow open triangle), and two-step phasing (blue open square) libraries are shown.

UCHIME2 was used to identify and remove chimeric sequences (Table S2A through C). Chimeric sequences (identified using the mock community 16S rRNA gene sequences as reference) accounted for about 11% of the raw joined sequences in Bm1 with non-phasing and one-step phasing, but only about 6.5% with two-step phasing (Fig. 1A). About 70% of these chimeric sequences were detected and removed by UCHIME2 using the Greengenes database as reference, regardless of amplification method used. About 30% of the chimeric sequences were not detected (Fig. 1A; Table S2A). Similar trends were observed in the forward and reverse reads (Table S2A). There were fewer chimeric sequences (~3%) in Bm2, which had a high abundance of low GC strains, compared to Bm1 (Fig. 1B; Table S2B). To rule out the possibility that the low chimera rate of Bm2 was due to lower similarities among the low-GC-content strains, we constructed a phylogenetic tree to visualize the similarities between the 33 mock community strains. Surprisingly, we found that low-GC-content strains tended to have more similar V4 regions when compared to the high-GC and medium-GC strains (Fig. S1). This was further supported by the higher GC contents of the chimeric reads than those of the non-chimeric reads in the Bm1 trimmed community (*P* < 0.001; Fig. S2). Bm3, with a high abundance of high-GC strains, had a similar percentage to Bm1 (Fig. 1C; Table S2C).

Quality trimming did not reduce the number of chimeric sequences for the joined sequences (Fig. 1D) but did slightly for the reverse and forward reads for the non-phasing and one-step phasing methods (Fig. 1E and F).

### Error rates

**Fig 2:**
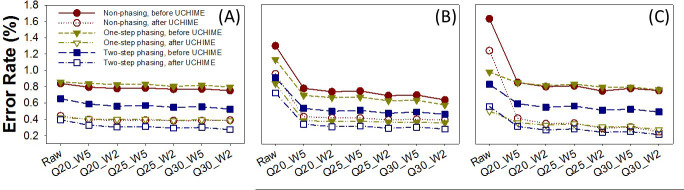
Sequence error rates for Bm1. (**A**) Forward sequences, (**B**) reverse sequences, and (**C**) joined sequences. Sequence error rates (only considering substitutions) were calculated by comparing the sequences to their mock community strain reference sequences for the raw, and quality trimmed (at stringencies from Q20-W5 through Q30-W2) sequences, for joint, forward, and reverse sequences. Sequence error rates were estimated before and after chimera removal by UCHIME using Greengenes core database for 16S rRNA gene sequences as reference sequences. The average sequence error rates before chimera removal (long dash line) for non-phasing libraries (dark red circle), one-step phasing (dark yellow triangle), and two-step phasing (blue square) libraries and after chimera removal (dotted line) for non-phasing libraries (dark red open circle), one-step phasing (dark yellow open triangle), and two-step phasing (blue open square) libraries are shown.

Sequence errors rates for all methods are shown in Table S3A through C; Fig. 2. The largest error rate for Bm1 was that of the non-phasing method raw reverse reads (1.63%; Fig. 2C). This rate was reduced after removing the detected chimera (1.24%; Fig. 2C) and after merging the forward and reverse reads (0.44%, without chimera; Fig. 2A). The two-step phasing method lowered the overall percent of sequencing errors (0.39%, without chimera; Fig. 2A). Quality trimming significantly reduced error rates as well. For example, the error rate for the two-step phasing joined sequences was 0.33% (chimera removed) after trimming (Q25-W5), which was further reduced to 0.27% with more stringent trimming (Q30-W2) (Fig. 2A). Similar error rates were observed with Bm3 (Table S3B), while Bm2 had much lower error rates (Table S3B), indicating the GC content of the sequences affected error rates.

### Operational taxonomic units or amplicon sequence variants

OTUs were estimated for joined sequences using UPARSE (Tables S4 and S5). For Bm1, most of the strains were recovered with only a few exceptions. One strain was missing from the two-step phasing library, and two strains were missing from non-phasing and one-step phasing libraries. UCHIME2 with a balanced mode identified only one chimera with two-step phasing and two with non-phasing and one-step phasing methods (66). Two-step phasing detected more contaminants (8 ± 3) and erroneous OTUs (14 ± 3), which could be true rare species, than non-phasing and one-step phasing methods due to the higher quality sequences generated from the two-step phasing. Although Bm2 and Bm3 were like Bm1 in number of OTU contaminants and erroneous sequences, there were no chimeras identified in Bm2, and fewer strains were detected in Bm2 and Bm3.

ASVs or (z)OTUs were also estimated for Bm3 with the two-step phasing method using DADA2, Deblur, UNOISE, and UCLUST. The number of ASVs detected by DADA2 (31 ± 5) and Deblur (34 ± 4) was close to the number of real mock community strains with very few artifacts, while the number of OTUs detected by UCLUST (110 ± 25) largely exceeded the number of mock community strains (Table S6A). However, DADA2 and Deblur missed more mock community strains (9 ± 2 and 8 ± 2, respectively), including one high-abundance strain (*Thermomicrobium roseum*, abundance 8.41%) (Table S6A). UCLUST (82 ± 25) and UNOISE (46 ± 8) had the most artifact (z)OTUs, most of which were chimeras (Table S6A). Erroneous (z)OTUs or ASVs accounted for 4%–20% of artifacts with all methods (Table S6A). UPARSE detected the most contaminants (9 ± 3), while DADA2 and Deblur detected only 1 ± 1 (Table S6A). The artifact composition for all methods was similar when using both sensitive and balanced modes, except that the proportion of chimeras increased and erroneous sequences decreased with sensitive mode, especially for UPARSE (Table S6B). A total of 69 contaminant ASVs or (z)OTUs were detected across all five methods. Twenty-five of the contaminants were archaea, all but one of which matched the 16S rDNA sequences of strains in an archaea mock community (65). Forty-four of the contaminants were bacteria, most of which matched 16S rDNA sequences of environmental samples in the NCBI database with a minimum identity of 94% (Table S7).

### Spurious sequences

Spurious sequences were defined as sequences in singletons, doubletons, and other unique OTUs. After trimming at Q20-W5, chimeric sequence removal, and OTU generation by UCLUST, the largest number of spurious sequences in Bm1 was detected with non-phasing (516), and the least occurred with two-step phasing (465) (Table S8A). Interestingly, Bm2 had the most spurious sequences when the two-step phasing method (284) was used, and the least when the non-phasing method (183) was used (Table S8B). Bm3 had similar numbers of spurious sequences (278–298) regardless of which amplification methods were used (Table S8C).

Almost all spurious sequences were singletons (97%–99%), which consisted of chimeric sequences (25%–75%), *E. coli* (0.7%–1.1%), or other contaminant strains (9%–32%) and erroneous sequences (16%–50%) (Table S8A through C). For the two-step phasing amplification method, its spurious sequences contained fewer chimeras, but relatively more contaminants and erroneous sequences, while its total number of spurious sequences was generally lower (Table S8A through C). Three singletons detected in Bm3 (one with one-step phasing, two with two-step phasing) were true sequences (Table S8C).

### Quantitation and bias

**Fig 3:**
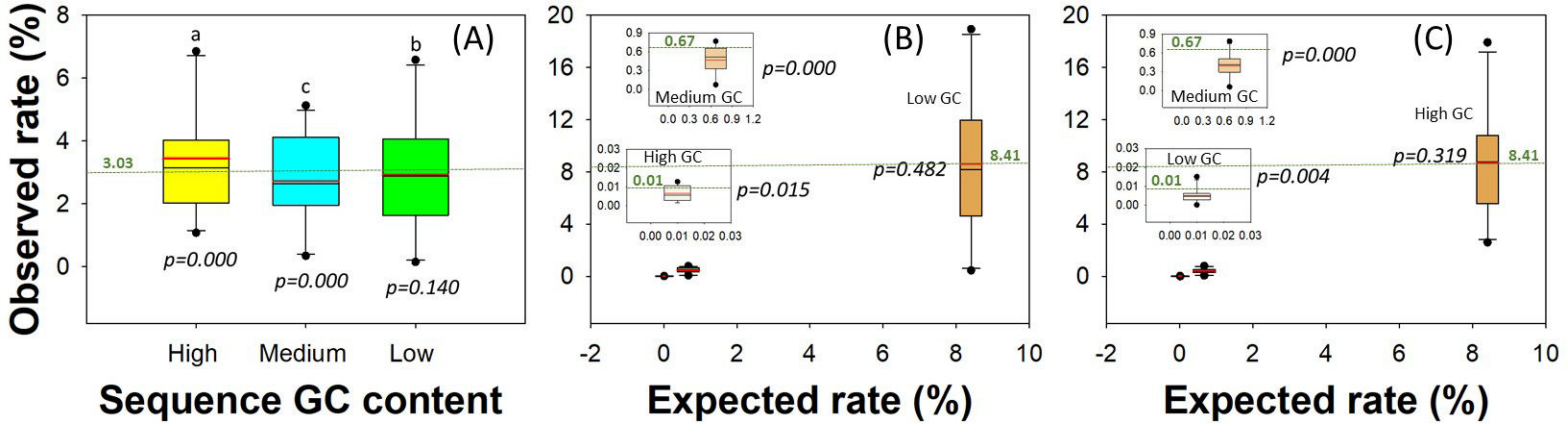
Effects of sequence GC content and input sequence abundances on mock community strain sequence recovery. The figure shows how mock community strain sequence GC content affected sequence recoveries in Bm1 with equal inputted abundances and high, medium, and low sequence GC content (**A**) and how inputted strain sequence abundances affected mock community strain sequence recoveries in Bm2 (**B**) and Bm3 (**C**), both with high, medium, and low inputted sequence abundances. Small plots within panels **B** and **C** show the observed recoveries of the strains with medium and low inputted abundances at a fine scale. The corresponding GC contents of Bm2 and Bm3 were indicated in the figures. The green lines and numbers in the major and small plots indicate the expected rates of even (3.03%), high (8.41%), medium (0.67%), and low (0.01%) input. Shown here are values of non-phasing method. Boxes and whiskers indicate quartiles, and red line indicate mean values. *P* values are for the comparisons between observed abundances and the expected. Lowercase letters (i.e., a, b, c) in panel **A** show the results of ANOVA and LSD tests to examine the significant differences among the observed abundances of high-, medium-, and low-GC-content strains.

Quantitative accuracy and sequencing biases were evaluated by comparing the observed and expected relative sequence abundances. The observed abundances were approximately fivefold lower to twofold higher than expected in Bm1 (Table S9A). Similar differences were observed for Bm2 (Table S9B) and Bm3 (Table S9C), except that one strain was not detected in Bm3 when the non-phasing method was used (Table S9C). The GC content and inputted abundance of strain sequences (expected) affected their observed rates (Fig. 3; Table 1). When using non-phasing method, it was found, for high-GC strains in equal (3.03%, Bm1) abundance community, the mean observed rate was higher than the expected (*P* < 0.001). Furthermore, it was significantly higher than those of both low- and medium-GC strains (Fig. 3A; Table 1). For low-GC strains with low input abundances (0.01%, Bm3; Fig. 3C) and medium-GC strains with medium (0.67%, Bm2 and Bm3; Fig. 3B and C) and equal (3.03%, Bm1; Fig. 3A) input abundances, the observed mean rates were lower than the expected (*P* < 0.01; Fig. 3B and C; Table 1). However, for high-GC strains with low input abundances (0.01%, Bm2; Fig. 3B), the mean observed abundance was lower than the expected (*P* < 0.05; Fig. 3B and C; Table 1). For low-GC strains with equal (3.03%, Bm1) and high (8.41, Bm2) input abundances, the differences between observed abundances and the expected were not significant (Table 1). These trends were also observed significantly in some cases but not in others when using one- or two-step phasing methods (Table 1).

**TABLE 1:** Comparisons of observed to expected strain rate among GC content, library preparation methods, and strain distribution in mock communities

Mock community			Bm1			Bm2			Bm3		
Group			1	2	3	1	2	3	1	2	3
GC content			Low	Medium	High	Low	Medium	High	Low	Medium	High
Expected rate (%)			3.03	3.03	3.03	8.41	0.67	0.01	0.01	0.67	8.41
Observed rate (%)	Non-phasing	Value	2.901	2.658	3.389	8.572	0.47	0.005	0.005	0.377	8.598
		sdtv	1.87	1.324	1.687	5.453	0.216	0.005	0.005	0.17	4.223
		*P* value^*a*^	0.140	0.000	0.000	0.482	0.000	0.015	0.004	0.000	0.319
		Significance^*b*^	b	c	a						
	One-step phasing	Value	2.665	3.035	3.229	8.515	0.505	0.011	0.006	0.407	8.577
		sdtv	1.217	1.453	0.764	3.966	0.242	0.003	0.005	0.174	1.997
		*P* value^*a*^	0.028	0.978	0.270	0.828	0.000	0.693	0.063	0.000	0.719
		Significance^*b*^	b	b	a						
	Two-step phasing	Value	3.163	2.754	3.073	8.561	0.464	0.009	0.012	0.493	8.517
		sdtv	1.545	1.738	1.177	4.194	0.249	0.003	0.006	0.282	3.66
		*P* value^*a*^	0.052	0.000	0.498	0.231	0.000	0.656	0.351	0.000	0.316
		Significance^*b*^	a	b	a						

^*a*^ *P* value from two-tail *t*-test against the expected value.

^*b*^ Lowercase letters (i.e., a, b, ab, and c) show the results of ANOVA and LSD tests to examine the significant differences.

Pearson correlation coefficients for Bm1 strains were 0.43 ± 0.05, 0.72 ± 0.12, and 0.76 ± 0.03 with non-phasing, one- and two-step phasing methods, respectively (Table 2), indicating a relative stronger positive correlation between the observed and expected results with the phasing methods. Similar results were obtained for Bm2 and Bm3 (Table 2).

**TABLE 2:** Pearson correlation coefficient between the observed and the theoretical abundances of mock community sequences

Methods	Mock community		
	Bm1	Bm2	Bm3
Non-phasing	0.4324 ± 0.0451 b^*a*^	0.7220 ± 0.0218 c	0.8554 ± 0.0126 b
One-step phasing	0.7175 ± 0.1192 a	0.8282 ± 0.0325 b	0.8935 ± 0.0492 a
Two-step phasing	0.7607 ± 0.0302 a	0.8461 ± 0.0163 a	0.8373 ± 0.0190 c

^*a*^ Lowercase letters (i.e., a, b, and c) following the value of the correlation coefficient in the table show the results of ANOVA and LSD tests to examine the significant differences among the methods.

**Fig 4:**
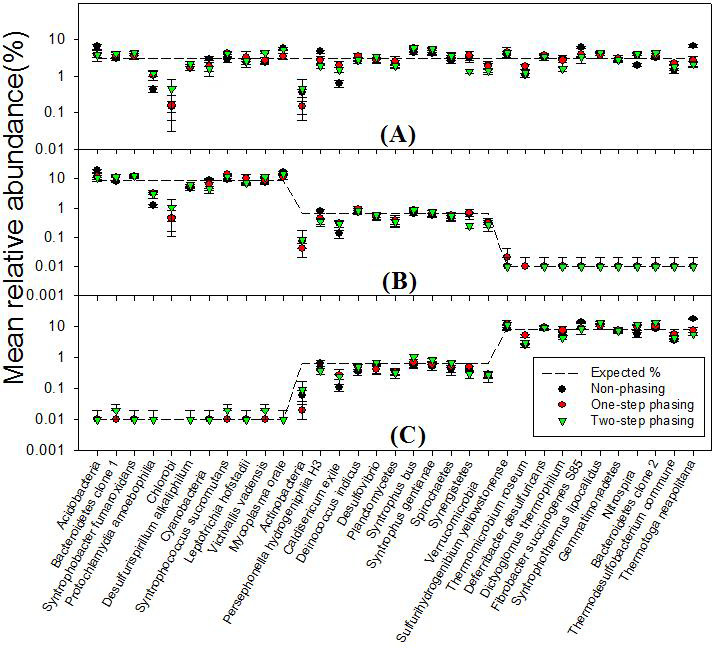
Relative abundance of strain sequences used to generate bacterial mock communities. (**A**) Bm1, (**B**) Bm2, (**C**) Bm3. Dashed line: theoretical relative abundance. The experimental mean relative abundances for non-phasing libraries (black circles), one-step phasing (red circles), and two-step phasing (green triangle) libraries are shown, and error bars indicate standard deviations for replicate samples.

**Fig 5:**
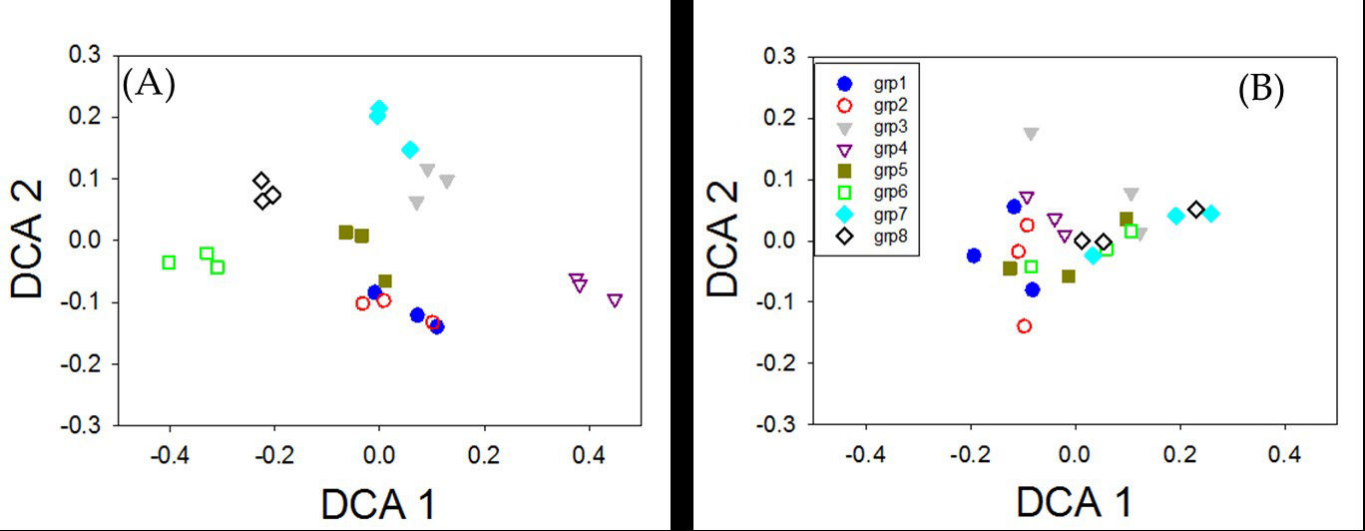
Detrended correspondence analysis (DCA) of the mock community 16S rRNA gene sequence data with one-step (**A**) and two-step (**B**) phasing methods showing community structural bias among different phasing primer groups with different length of spacers. Mock community libraries with phasing primer groups 1 (blue circle, forward spacers/reverse spacers [the same for other groups], 0/7), 2 (red open circle, 1/6), 3 (gray triangle, 2/5), 4 (open purple triangle, 3/4), 5 (dark yellow square, 4/3), 6 (open green square, 5/2), 7 (light blue diamond, 6/1), and 8 (open black diamond, 7/0) with triplicate were shown.

Abundance biases were observed for specific strains (Fig. 4). For example, several strains, including those with low(*Protochlamydia amoebophilia*, *Chlorobi*), moderate (*Actinobacteria*, and *Caldisericum*), and high (*Thermomicrobium roseum* and *Thermodesulfobacterium commune*) GC content, had consistently low recovery, regardless of how abundant they were or which amplification method was used (Fig. 4). We found that the low recovery of these strains was due to mismatching to the forward PCR primer (data not shown). A few other strains had consistently high recoveries when in moderate to high abundance but not in low abundance (*Acidobacteria*, *Mycoplasma orale* [low GC]) (Fig. 4). In addition, primer groups displayed some biases based on amplification method used (Fig. 5). Ordination plots of the sequencing results showed a clear separation of replicated libraries among the primer groups when one-step phasing was used (Fig. 5A) but no separation with two-step phasing (Fig. 5B).

## DISCUSSION

Sequence errors generated by MiSeq amplicon sequencing are generally due to low sequence quality, chimeric sequences, and errors generated during PCR amplification. Some errors may be related to specific sequence regions, such as inverted repeats or repeated bases (GGG) (65). Errors with low-quality scores are usually removed during quality trimming at Q20-W5. Although increasing the stringency of quality score-based trimming can remove additional errors, it is at the expense of sequencing depth (7). Combining forward and reverse reads could also decrease error rates which is consistent with Kozich’s report, in which a ΔQ strategy was used to remove unreliable consensuses (11). However, dNTP misincorporation errors generated during PCR amplification, which can be a major source of error in quality trimmed sequences, cannot be identified based on base quality score. Kozich used a pre-clustering strategy that a rare sequence with low abundance was removed and its abundance was added to a sequence more abundant and had difference less than 1 nt per 100 nt with the less abundant sequence, which further reduced errors. This strategy could remove some errors generated during PCR. We observed that some sequence positions were more prone to error than others. This could be related to specific sequence structures, as reported by other researchers (65). However, the error rates in these sequence positions were much lower than the detection criterion (30%) (65) and accounted for only a very small portion of the remaining error rate (data not shown) in this study. In addition, we found that sequence reads generated from high-GC-content mock communities (Bm1 and 3) had higher error rates compared to those from the low-GC-content mock community (Bm2), which was further supported by the error rates of the three strain clusters with different GC content of Bm1 (Fig. S3). Sze et al. found that the error rate increased with PCR cycles (12). In this study, we found that two-step PCR lowered the error rate that could be due to the second PCR refreshed enzyme and other reagents.

We found that UCHIME with the Greengenes sequence database as reference could not remove all chimeric sequences, usually missing about 30% of the chimeric sequences. We also found that the most prevalent spurious sequences detected were chimeras. The use of two-step PCR reduced the number of chimeric sequences by half because two separate PCRs were used, each having not more than 20 (67) PCR cycles and fresh reagents, which was consistent with Sze et al.’s report, in which it was found that the chimera rate increased with PCR cycles (12). We observed that several other factors influenced chimeric sequence formation, including GC content and the distribution of strains in the community being sequenced. Low-GC DNA sequences generated fewer chimeric sequences as observed with the high-abundance, low-GC strains in Bm2. This observation may be due to the lower binding free energy of DNA duplexes (68), which makes incomplete PCR products with a low GC content less likely to bind to the template DNA in subsequent PCR cycles, thereby reducing the chance of chimera formation. In addition, community DNA with one or more dominant strains, rather than many similar abundant strains, resulted in a lower chimeric sequence rate. This is because a chimeric sequence formed within a single strain population is a self-chimeric sequence and is thus no different from a normal PCR product. So, if dominant strains are present, there would be a higher number of sequences from only a few strains, making self-chimera more likely. Real community DNA has a much higher diversity than the mock communities used in this study, so it would be expected to have a higher chimeric sequence rate than observed here. A more thorough understanding of the factors contributing to chimera formation may help in the development of algorithms better able to detect chimeric sequences.

A common practice to clean up OTU tables is to remove potentially spurious sequences before downstream analyses, an approach partially supported by the results of this study. Most of the spurious sequences (25%–75%) were identified as chimeric sequences. About 10% of the spurious sequences were contaminants from reagents used for PCR amplification, 16S rRNA gene cloning (69, 70), and the laboratory environment, which was confirmed by the template free control 16S rRNA gene sequencing analysis (Table S10A through C). Thus, preventing contamination during preparation and amplification steps is extremely important. A fraction (16%–34%) of the spurious sequences were erroneous due to mismatching during PCR amplification and sequencing. Two-step phasing method resulted in more effective sequences (7), fewer chimeras, and fewer spurious sequences, primarily due to a lower chimera rate and higher sequence quality. Of its spurious sequences, there were still fewer chimeras; there was a relatively higher proportion of contaminants and erroneous sequences. It is worth noting that contaminants and sequences with errors introduced during PCR amplification could be considered genuine rare sequences, indicating that two-step phasing allowed for the retrieval of a greater number of rare sequences. One or two presumed spurious sequences were identified as true sequences in some of the mock communities. Using standard practices, these sequences would be removed, thus missing what could be true rare species. Even with the limited community size of our mock community and the relatively high abundance (0.01%) of our “low-abundance” strains, the low-abundance strains appeared to be under-sequenced. In real communities, with much higher diversities and more members at very low abundance, the likelihood that presumed spurious sequences would include true sequences would be much higher. As such, there should be a balance between restrictive quality trimming and preserving less abundant (z)OTUs or ASVs.

The remaining sequencing artifacts were erroneous sequences that had high sequence quality scores and originated from one of the PCR amplification steps. OTU sequence error rates were significantly negatively correlated with OTU sequence abundance (Fig. S4). Thus, the remaining errors were mostly restricted to low-abundance OTU sequences. Using DADA2 and Deblur to process amplicon sequences resulted in fewer spurious ASVs due to more optimal removal of erroneous sequences.

Another important consideration for microbial detection is the quantitative capability of the amplicon sequencing approach. In this study, we found that the quantitative capability of targe gene amplicon sequencing is limited. Although the observed and expected abundances of each strain showed reasonable correlation, the differences were relatively large, averaging two- to sixfold. Higher abundance strains generally had observed abundances that were close to the expected abundance, whereas low-abundance strains consistently had observed abundances lower than expected when non-phasing method was used. These findings suggest that rare species’ presence in real communities is likely to be underestimated. It is worth noting that the lowest concentration evaluated in this study was 0.01%, which was much higher than the typical concentration observed in nature. As a result, the quantitative capability of amplicon sequencing is likely poorer when applied to real community samples. These findings are consistent with what we observed previously with 454 amplicon sequencing (60). In this study, the GC content of the sequences also affected recovery rates that low-GC sequences with low input abundance and medium-GC sequences with equal and medium input abundances exhibited relatively low recoveries and high-GC sequences with equal input abundances exhibited relatively high recoveries using non-phasing methods. Using one- and two-step phasing methods, these biases were mitigated. Sequencing bias was also observed for PCR primer pairs, which was partially due to mismatches in forward primer sequences of mock community strain 16S rRNA sequences and was consistent with a previous report (71), and for spacer length differences. Spacer bias was only observed with one-step phasing, indicating the need for a two-step PCR.

The findings of this study, which were based on sequencing of the V4 region of 16S rRNA genes, may provide valuable insights for other targeted gene amplicon sequencing, including those targeting functional genes. However, it is important to note that the secondary structure and frequency of interspecific complementarity required for chimera formation may differ significantly for other classes of genes compared to 16S rRNA. Furthermore, different classes of genes may have GC contents outside of the range investigated in this study. Therefore, further investigation of different functional genes is necessary to fully understand the implications of these findings for other types of sequencing assays.

PCR methods incorporating phasing primers improved sequencing quality by increasing the cluster density, passing filter and Q30 percentages, and lowering the raw sequence error rate, especially toward the end of a run. This, in turn, increased the number of qualified sequences reads after quality trimming, consistent with previous reports (7). A method that combines shotgun whole genome sequencing with amplicon sequencing or, alternatively, adding more (15%) Phix can also overcome the phasing problem in MiSeq sequencing when phasing primers are not available. In addition, the two-step, phasing PCR reduced the number of chimeric sequences by as much as 50%, although, based on the results using UPARSE, two-step phasing resulted in more contaminants and erroneous OTUs, which may be due to there being more of these sequences having higher quality and fewer chimeras remaining. The two-step PCR amplification also eliminated sequencing biases observed from spacer differences in the phasing, one-step PCR. These findings provide further evidence of improved sequencing quality when using the phasing, two-step PCR method.

Although we could not draw a conclusion for which sequence processing method performed best, we did see specific strengths for each method. UPARSE detected a higher number of OTUs, recovering most of the mock community strains and missing only 3 low and 1 medium abundance strains, indicating a high recovery of the mock community diversity. In addition, UPARSE detected the most contaminants, particularly strains in an archaea mock community that was created together with the bacterial mock communities used in this study. If these archaea strains and other contaminants were considered true or putatively positive strains, UPARSE detected the most low-abundance stains, indicating that it may be better at detecting true rare species in real environmental samples. UPARSE also had fewer chimeric OTUs, indicating that it is good at removing chimera. UCLUST had the most artifacts, over twice of the mock community strains; moreover, chimera accounted for the largest fraction of artifacts compared to the other methods. UNOISE performed similarly to UCLUST. There were about one and half times the number of artifacts as mock community strains with UNOISE, and chimeras made up 56% of its artifacts. For UNOISE and UCLUST, chimera would be of significant concern with real community samples because chimeric sequences would be more difficult to detect. So, these methods could overestimate the diversity of real microbial communities. Both DADA2 and Deblur had very few artifacts but missed many of the low abundance mock community strains, and both detected very few contaminants, specifically strains in the contaminant archaea mock community, which could be with too restrictive sequence processing settings. Since real microbial communities have much higher diversity with a large percentage of rare species, the abundance of which would be much lower than what was used in the mock communities, they would likely not be recovered. Thus, these two methods could significantly underestimate the diversity of communities in environmental samples. Although the number of ASVs detected by DADA2 and Deblur was close to the number of mock community strains, there were still artifacts, including chimeras and erroneous ASVs. Thus, artifacts are a common and unavoidable issue for all five methods. Great caution should be taken when selecting appropriate methods for sequence analysis, which is questions and objectives dependent. For example, if rare species are a focus, then, DADA2 and Deblur would not be good choices. Sequencing errors, artifacts, biases, and inherent technical variations associated with sequencing and processing steps are unavoidable. As such, sequence data are best used for relative comparisons across different conditions or treatments (8, 60, 72) so that the effects of technical variation on the ﬁnal experimental outcomes can be canceled out (8).

### Conclusions

This study rigorously investigated the origins and influential determinants of artifacts and methodological biases in target gene amplicon sequencing with mock communities, featuring diverse bacterial strains’ 16S rRNA genes, leading to interesting findings. First, the research unveiled that chimeric sequence significantly contributed to sequencing artifacts, constituting up to over 10% of raw sequences, of which a third persisted post-UCHIME treatment using the GreenGene database as reference. Notably, spurious sequences, particularly singletons and doubletons, were predominated by chimeras. Moreover, a substantial association emerged between chimeric sequence occurrence and the GC content of targeted sequences. Sequences with lower GC content demonstrated diminished chimera formation rates. Second, while most errors were linked to sequence quality, a subset of errors arose during PCR amplification, persisting after quality trimming and concentrating within rare OTUs/ASVs. GC content of target sequences exhibited a direct relationship with error rates. Third, target sequence GC content and input abundance profoundly influenced mock community strain recovery, with elevated GC content and higher input abundance enhancing recovery rates, further entailing interactive effects. Fourth, the quantitative capacity of target gene amplicon sequencing displayed notable limitations, characterized by substantial recovery variations and weak correlation between anticipated (input) and observed strain abundances. Fifth, biases stemming from primer affinity were identified. Application of a two-step phasing strategy during library preparation and sequencing yielded multifaceted benefits, reducing chimeric sequences, elevating sequence quality, augmenting effective sequence yields, enabling rare sequence recovery, and mitigating the impacts of target sequence GC content and abundances on mock community strain retrieval. This strategy effectively counteracted biases introduced by target gene primers and barcoded primers. Comparative assessment of diverse amplicon sequence processing pipelines, including DADA2, Deblur, UNOISE, UCLUST, and UPARSE, highlighted distinct merits and demerits. While DADA2 and Deblur exhibited fewer artifacts, they incurred greater strain losses. In contrast, UCLUST and UNOISE yielded increased artifacts but recovered more mock community strains. UPARSE demonstrated superior efficacy in minimizing artifacts and mock community strain loss. Consequently, for mitigating chimeric sequences, errors, and methodological biases, adoption of the two-step phasing approach during PCR library preparation and sequencing was recommended. The selection of an appropriate sequence processing pipeline was contingent upon scientific objectives, favoring UPARSE with DADA2 as a suitable alternative when rare species detection was of secondary concern. The study’s insights into the influence of target sequence GC content, abundances, and associated factors on chimeric sequence formation and error rates hold promise for advancing enhanced approaches in sequence generation and processing methodologies.

## MATERIALS AND METHODS

### Mock community DNA

The mock communities used in this study were reported previously (71). Briefly, 16S rRNA gene sequences from 33 bacterial strains, belonging to 27 different phyla, were used to construct the mock communities (Table S11; File S1) (71). Clones of near full-length 16S rRNA gene fragments were generated using PGEM-T Easy Vector II system (Promega, Inc., Madison, WI). The plasmid concentrations were quantified in triplicate using Quant-iT dsDNA assay kit (Invitrogen, Carlsbad, CA) on a Nanodrop 3300 (Thermo Scientific, Wilmington, DE). The sequences were divided into three clusters mostly (with a few exceptions) based on GC content within the V3–V5 region of the 16S rRNA genes and, considering the strain diversity at the phylum level, the GC content of V4 region: low (51.16% ± 2.1%), moderate (55.1% ± 1.8%), and high (59.3% ± 3.3%), with 11 in each cluster to assess whether overall community GC content of the target sequences causes biases. The sequences of the three clusters were then distributed into three mock communities in equal (3.03%) abundance in bacterial mock community 1 (Bm1) and in a combination of low (0.01%), moderate (0.67%), and high (8.41%) abundance in two different allotments (Bm2 and Bm3) (Table S11). All mock communities had a 16S rRNA gene concentration of 10^9^ copies/µL.

### PCR library preparation

Primer pair 515F (5′-GTGCCAGCMGCCGCGGTAA-3′) and 806R (5′-GGACTACHVGGGTWTCTAAT-3′) (2) were used to amplify the V4 hypervariable region of the bacterial 16S rRNA genes. Three strategies were used for PCR library preparation: one-step PCR with non-phasing primers (non-phasing), one-step PCR with phasing primers (one-step phasing), and two-step PCR with phasing primers (two-step phasing). For the non-phasing method, both forward and reverse primers contained the Illumina adapter, pad, and linker sequences (Table S12A and B). The reverse primers also contained a barcode sequence (12-mer) between the Illumina adapter and pad sequences (Table S12B) (1). For both one-step and two-step phasing methods, phasing primers (Table S12C through E) were used for library generation, and for two-step phasing, the target-only primers were used in the first PCR (7). Both forward and reverse phasing primers contained the Illumina adapter, the sequencing primer, a spacer, and the target gene primer; a 12-mer barcode sequence was on the reverse primer between the Illumina adapter and the sequencing primer.

For the non-phasing method, 24 libraries with unique barcodes (Table S12A and B) were generated for each of the mock communities as technical replicates. The content of each amplification reaction and the thermal cycling conditions were described previously (7). Each library had triplicate reactions. Following amplification, 2 µL of PCR product from each reaction was used for agarose gel (1%) electrophoresis to confirm amplification. Each library was generated by pooling the triplicate PCRs and then quantified with PicoGreen.

For the one- and two-step phasing methods, we generated 24 libraries with unique barcodes for each mock community using phasing primers. To achieve this, we selected one primer from each of the eight forward primers and three primers with unique barcodes from each of the eight reverse primer groups to ensure that the primer pairs had different spacer lengths for the forward and reverse primers, while maintaining a total spacer length of 7. This enabled us to analyze the bias caused by different spacer combinations of forward and reverse primers (Table S12C through E) (7). PCR amplification with the one-step phasing method followed the same procedure as that of the non-phasing method (7). For the two-step PCR, target-only primers and 10 cycles of amplification were used for the first PCR, and then phasing primers and 20 cycles of amplification were used for the second PCR (7).

### Sequencing

A 200-ng aliquot of PCR product from each library was pooled, purified using a QIAquick Gel Extraction Kit (QIAGEN Sciences, Germantown, MD, USA), and then quantified with PicoGreen for one MiSeq run (7). The sample library was prepared for sequencing following the MiSeq Reagent Kit Preparation Guide (Illumina, San Diego, CA, USA) as described previously (7). The mock community libraries for each of the three PCR methods (each had 3 × 24 = 72 libraries) were pooled and sequenced separately. The detailed experiment design is shown in Table 3.

**TABLE 3:** Details of experiment design

Methods	Non-phasing	One-step phasing	Two-step phasing
PCR steps	One	One	Two
First step PCR primer	Barcode primer	Barcode primer	Target gene primer
Second step PCR primer	n/a^*a*^	n/a	Barcode primer
Spacers in barcode primer	None	0–7	0–7
First step PCR cycles	35	35	10
Second step PCR cycles	n/a	n/a	20
Barcode primer groups	1	8	8
Replicates of each primer group	24	3	3
Total replicates	24	24	24
Number of mock communities	3	3	3
Number of libraries	72	72	72
MiSeq run	1	1	1

^*a*^ n/a, not applicable.

### Sequence data processing and statistics

Raw sequence quality check and data preparation were performed as previously described (7). To identify the origins of the reads, BLAST (73) was used to search against the reference sequences, where the closest match was recorded for each read. Btrim was used for quality trimming of both forward and reverse reads based on the sequence quality score (74). To evaluate the effect of trimming strategies, sequences were trimmed if the average quality score in a window of 5 or 2 bases (W5 or W2) was not continuously equal or lagger than 20, 25, or 30 (Q20, Q25, or Q30). Sequences that were less than 200 bases or contained undetermined bases, N’s, were removed. FLASH v1.2.5 (75) was used to merge paired end reads with sufficient overlap (minimum 20 bases) into full-length sequences. Reads that could not be joined were removed.

Chimeric sequences were identified based on predictions from the UCHIME2 algorithm (66) in USEARCH using 16S rRNA sequences from either the Greengenes core database (76) or the mock community strains as references. All chimeric sequences predicted using the mock community sequences were true chimeras. Those predicted by the Greengenes database were predicted (or detected) chimeras that would be removed during sequence quality filtering. The undetected chimeric sequences were those present after subtracting the predicted from the true chimeras. All mock community strains have at least 80% similarity to one 16S rRNA reference sequence in Greengenes core database, seven of them have identical matches.

To estimate the error rate, the sequence reads (forward, reverse, or merged) were searched against the reference sequences and aligned using the “usearch_global” command provided by USEARCH (66). Alignment to the best hit reference sequence was used to determine the number of mismatches, insertions, and deletions at each base position. The error rate was estimated using the total number of errors divided by the total valid base number for each base position and for each sequence. Sequence error rates were estimated for the raw reads and merged reads, after quality trimming, and before and after chimeric sequence removal.

The OTUs from all mock communities were clustered using UPARSE (77). Additionally, ASVs or (z)OTUs from Bm3 were clustered using DADA2 (78), Deblur, UCLUST, and UNOISE. This was done to compare the different methods in terms of the total number of ASVs/(z)OTUs detected, artifact removal, artifact composition, and rare species recovery. DADA2 and Deblur were performed in QIIME2 (https://qiime2.org); UCLUST, UNOISE3, and UPARSE were performed in USEARCH (79). Representative sequences generated from each method were classified using sequences of the 33 mock community bacteria. A maximum error of 7 of 253 bp was allowed for generation of OTUs with UCLUST and UPARSE at a similarity threshold of 97%. No errors were allowed with the other methods. To control variation due to an unequal number of sequences detected across libraries, sequence resampling was performed prior to any processing for each library based on the library with the fewest number of sequences. Resampling was accomplished by randomly drawing sequences from the original pool until the selected rarefying level was reached. Spurious sequences or sequences in unique OTUs, which was defined as OTUs present in only one technical replicate across all mock community libraries, containing one (singletons), two (doubletons) or more than two (other unique OTUs) sequences were identified to investigate their compositions. To identify the source of false-positive sequences (artifacts), we classified the sequences into chimera, contaminated, and erroneous sequences. Chimera detection was done as described above using the 16S rRNA V4 region sequences of the 33 mock community bacteria strains as the reference database. Both sensitive mode (at the expense of a high false-positive rate) and balanced mode, which seeks to minimize the overall error rate, were used for chimera detection. To distinguish contaminant and erroneous sequences, we used a BLAST search for all false-positive sequences against the NCBI database and a database comprised of the 33 mock community bacteria strains. Non-chimera false-positive sequences that matched to the NCBI database with a greater than 70% identity while not matching to the mock community database were defined as contaminant strains. Erroneous sequences were defined as non-chimera false-positive sequences that matched to the mock community database with a greater than 70% identity.

## Data Availability

The mock community sequencing data are available at NCBI under accession number PRJNA576519.

## SUPPLEMENTAL MATERIAL

File S1 msystems.01025-23-s0001.docx Bacterial mock community strain V4 sequences.

Supplemental figures msystems.01025-23-s0002.docx Figures S1-S4.

Supplemental materials msystems.01025-23-s0003.docx List of the supplemental files and legends.

Supplemental tables, set 1 msystems.01025-23-s0004.docx Tables S1-S8 and S11.

Supplemental tables, set 2 msystems.01025-23-s0005.xlsx Table S10.

Supplemental tables, set 3 msystems.01025-23-s0006.xlsx Tables S9 and S12.

ASM does not own the copyrights to Supplemental Material that may be linked to, or accessed through, an article. The authors have granted ASM a non-exclusive, world-wide license to publish the Supplemental Material files. Please contact the corresponding author directly for reuse.
