# Supplementary material for: Effects of error, chimera, bias, and GC content on the accuracy of amplicon sequencing
Source: mSystems. 2023 Dec 1;8(6):e01025-23. doi: 10.1128/msystems.01025-23 (PMC10734440; doi:10.1128/msystems.01025-23)
Supplement: File S1 — Bacterial mock community strain V4 sequences. [file msystems.01025-23-s0001.docx]

>JQ346769.1 acidobacteria_jq346769

TACGTAGGGAGCAAGCGTTGTTCGGATTTACTGGGCGTAAAGGGCGCGTAGGCGGCGCGACAAGTCACTTGTGAAATCTC

CGGGCTTAACTCGGAACGGCCAAGTGAAACTGTCATGCTAGAGTGCAGAAGGGGCAATCGGAATTCTTGGTGTAGCGGTG

AAATGCGTAGATATCAAGAGGAACACCTGAGGTGAAGACGGGTTGCTGGGCTGACACTGACGCTGAGGCGCGAAAGCCAG

GGGAGCAAACGGG

>JQ346771.1 actinobacterium_jq346771

TACATAGGCTTCAAGCGTTGTCCGGATTTATTGGGCGTAAAGAGTTCGTAGGCGGTCGAGTAAGTCGGGTGTGAAAATTC

TGGGCTCAACCCAGAGACGCCACCCGATACTGCTTAACTTGAGTTCGATAGGGGAGTGGGGAATTCCTAGTGTAGCGGTG

AAATGCGCAGATATTAGGAGGAACACCGGTGGCGAAGGCGCCACTCTGGATCGACACTGACGCTGAGGAACGAAAGCATG

GGTAGCAAACAGG

>JQ346767.1 bacteroidetes_clone1_jq346767

TACGGAGGGTGCAAGCGTTGTCCGGATTTATTGGGTTTAAAGGGTGCGCAGGTGGTTTATTAAGTCAGTGGTGAAAGACG

GTCGCTCAACGATTGCAGTGCCATTGAAACTAGTAGACTTGAGTAAAGTAGAGGTGGGCGGAATTGATAGTGTAGCGGTG

AAATGCATAGATATTATCAAGAACTCCAATTGCGTAGGCAGCTCACTTGGCTTTTACTGACACTCATGCACGAAAGTGTG

GGTATCAAACAGG

>JQ346770.1 bacteroidetes_clone2_jq346770

TACGGAGGATCCGAGCGTTATCCGGATTTATTGGGTTTAAAGGGTGCGCAGGCGGCATGTTAAGTCGGCGGTGAAATTTT

GCAGCTCAACTGTAAAAGAGCCTTCGAAACTGGCAAGCTTGAGTGTGGATGAAGTAGGCGGAATTTGTGGTGTAGCGGTG

AAATGCATAGATATCACAAAGAACACCGATTGCGCAGGCAGCTTACTAAACCATAACTGACGCTCATGCACGAAGGCGTG

GGGATCAAACAGG

>JQ346734.1 caldisericum_exile_jq346734

AACGTAGGGGGCGAGCGTTATCCGGAATCACTGGGTGTAAAGGGTACGCAGGCGGACTTGTAAGTTGGATGTAAAAACTC

AGGGCTCAACCTTGAGCTTGCATTCAATACTACAAGTCTAGAGAGCAGGAGAGGGGAGTGGAATTCCCGGTGTAGGGGTA

AAATCCGTAGATATCGGGAGGAACACCAGTGGCGAAGGCGGCTCCCTGGCCTGTCTCTGACGCTCAGGTACGAAAGCGTG

GGGAGCAAACAGG

>JQ346768.1 chlorobi_jq346768

TACAGGGGTGGCAAGCGTTGTCCGGATTTACTGGGTGTAAAGGGTGCGCAGGCGGGCTCATAAGTCGGGGGTTAAATCCA

TGTGCTTAACACATGCATGGCTTCCGATACTGTGAGTCTAGAGTCTCGAAGAGGAAGATGGAATTTCCGGTGTAACGGTG

GAATGTGTAGATATCGGAAAGAACACCAGTGGCGAAGGCAGTCTTCTGGTCGAGAACTGACGCTCAGGCACGAAAGCGTG

GGGAGCAAACAGG

>JQ346766.1 cyanobacterium_jq346766

GACGGAGGATGCAAGTGTTATCCGGAATCACTGGGCGTAAAGCGTCTGTAGGTGGTTTAATAAGTCAACTGTTAAATCTT

GAGGCTCAACTTCAAAATCGCAGTCGAAACTATTAGACTAGAGTATAGTAGAGGTAAAGGGAATTTCCAGTGGAGCGGTG

AAATGCGTAGATATTGGAAAGAACACCGATGGCGAAAGCACTTTACTGGGCTATTACTAACACTCAGAGACGAAAGCTAG

GGTAGCAAATGGG

>JQ346740.1 deferribacter_desulfuricans_jq346740

TACGGAGGGGGCGAGCGTTGTTCGGAGTCACTGGGCGTAAAGCGCACGTAGGCGGTGCGGTAAGTCTGGGGTCAAAGGCT

ACGGCTCAACCGTAGTAAGGCCTCAGATACTATCGTGCTAGAGTGCCGGAGAGGGTAGCGGAATTCCCTGTGTAGCGGTG

AAATGCGTAGATATAGGGAGGAACACCGGTTGCGAAGGCGGCTACCTGGCCGGTGACTGACGCTGAGGTGCGAGAGCGTG

GGGAGCAAACAGG

>JQ346735.1 deinococcus_indicus_jq346735

TACGGAGGGTGCAAGCGTTACCCGGAATCACTGGGCGTAAAGGGCGTGTAGGCGGGATGGTAAGTCTGGTTTTAAAGACT

GCGGCTCAACCGCAGGGATGGACTGGATACTGGCATTCTTGACCTCTGGAGAGAGAACTGGAATTCCTGGTGTAGCGGTG

GAATGCGTAGATACCAGGAGGAACACCAATGGCGAAGGCAGGTTCTTGGACAGAAGGTGACGCTGAGGCGCGAAAGTGTG

GGGAGCGAACCGG

>AJ786059.1 desulfovibrio_aj786059

TACGGAGGGTGCGAGCGTTAATCGGAATCACTGGGCGTAAAGCGTGCGTAGGCGGCGCTTCAAGTCAGACGTGAAAGCCC

TCGGCTCAACCGAGGAATTGCGTTTGAAACTGAAGTGCTAGAGTCTCGGAGAGGTTGGCGGAATTCCAGGTGTAGGAGTG

AAATCCGTAGATATCTGGAGGAACACCGGTGGCGAAGGCGGCCAACTGGACGAGAACTGACGCTGAGGTACGAAAGCGTG

GGTAGCAAACAGG

>JQ346730.1 desulfurispirillum_alkaliphilum_jq346730

TACGGATGGAGCAAGCGTTGTCCGGAATCATTGGGCGTAAAGGGTGCGTAGGCGGTTTTTTAAGTCTGCATTGTAAGTTC

AGTGCTTAACGCTGAAATTGGTGCGGAAACTGGAAGACTTGAGTACTGTAGGGGAAAGCGGAATGCCCTGTGTAGAGGTG

AAATTCGTAGATATAGGGTGGAACATCAAAAGCGAAGGCAGCTTTCTGGGCAGTAACTGACGCTGAGGCACGAAAGCGTG

GGGAGCAAACAGG

>JQ346741.1 dictyoglomus_thermophilum_jq346741

GACGTAGGGGGCGAGCGTTGTCCGGATTTACTGGGCGTAAAGGGCGTGTAGGCGGCTTAGCAAGTCAGATGTGGAAGCCC

TGAGCTCAACTCAGGGAGGTCATCTGATACTGCTAAGCTAGAGGGCAGGAGAGGAGAGCGGAACTTCCGGTGTAGCGGTG

AAATGCGTAGATATCGGAAGGAACGCCGGTGGCGAAGGCGGCTCTCTGGACTGACCCTGACGCTGAGGCGCGAAAGCTAG

GGGAGCGAACGGG

>JQ346742.1 fibrobacter_succinogenes_jq346742

TACGAGGGGTGCAAGCGTTGTTCGGAATTACTGGGCGTAAAGGGAGCGTAGGCGGAGATTCAAGCGGATTGTACAATCCC

GGGGCCCAACCCCGGCTCTGTAGTCCGAACTGGATCTCTTGGATAGTTCAGGGGCAGGCGGAATTCCTGGTGTAGCGGTG

GAATGCGTAGAGATCAGGAAGAACACCGATGGCGAAGGCAGCCTGCTGGGGACTTATCGACGCTGAGGCTCGAAAGTGCG

GGTAGCAAACAGG

>JQ346776.1 gemmatimonadetes_jq346776

TACAGAGGGTGCGAGCGTTGTCCGGAATCACTGGGCGTAAAGGGCGCGTAGGCGGCCGATCAAGTGTGTGGTGAAAGCCC

GGGGCTCAACCCCGGGTCTGCCGTGCAAACTGGTTGGCTTGAGCACTGTAGAGGCAGGTGGAATTCCGGGTGTAGCGGTG

GAATGCGTAGAGATCCGGAAGAACACCGGTGGCGAAGGCGGCCTGCTGGGCAGTAGCTGACGCTGAGGCGCGACAGCGTG

GGGAGCAAACGGG

>JQ346732.1 leptotrichia_hofstadii_jq346732

TACGTATGTCGCAAGCGTTATCCGGAATTATTGGGCATAAAGGGCATCTAGGCGGCCAGGCAAGTCTGGGGTGAAAACTT

GCGGCTCAACCGCAAGCCTGCCCTGGAAACTGCCTGGCTAGAGTGCTGGAGAGGTGGACGGAACTGCACGAGTAGAGGTG

AAATTCGTAGATATGTGCAGGAATGCCGATGATGAAGATAGTTCACTGGACGGCAACTGACGCTGAAGTGCGAAAGCTGG

GGGAGCAAACAGG

>JQ346727.1 Mycoplasma orale culture DSM:19105 16S ribosomal RNA gene, partial sequence

TACATAGGTCGCAAGCGTTATCCGGAATTATTGGGCGTAAAGCGTTCGTAGGCTGTTTATTAAGTCTGGAGTCAAATCCC

AGGGCTCAACCCTGGCTCGCTTTGGATACTGGTAAACTAGAGTTAGATAGAGGTAAGCGGAATTCCATGTGGAGCGGTGA

AATGCGTAGATATATGGAAGAACACCAAAGGCGAAGGCAGCTTACTGGGTCTATACTGACGCTGAGGGACGAAAGCGTGG

GGAGCAAACAGG

>JQ346777.1 nitrospira_jq346777

TACGAAGGTGGCAAGCGTTGTTCGGATTCACTGGGCGTACAGGGAGCGTAGGCGGTTGGGTAAGCCCTCCGTGAAATCTC

CGGGCCTAACCCGGAAAGTGCAGAGGGGACTGCTCAGCTAGAGGATGGGAGAGGAGCGCGGAATTCCCGGTGTAGCGGTG

AAATGCGTAGAGATCGGGAGGAAGGCCGGTGGCGAAGGCGGCGCTCTGGAACATTTCTGACGCTGAGGCTCGAAAGCGTG

GGGAGCAAACAGG

>JQ346733.1 persephonella_hydrogeniphila_jq346733

TACGTAGGTCCCGAACGTTGCGCGAAATTACTGGGCGTAAAGGGTCCGTAGGCGGTCTGGTAAGTGGAAGGTGAAAGCCT

GGGGCTCAACTCCAGAATTGCCTTCCAAACTGCCGGACTTGAGGCAGGGAGAGGTCGGCGGAATTCCCGGTGTAGCGGTG

AAATGCGTAGATATCGGGAGGAACACCAGTGGCGAAGGCGGCCGACTGGAACTGTCCTGACGCTGAGGGACGAAAGCCAG

GGGAGCGAACCGG

>JQ346772.1 planctomycete_jq346772

TACGAACCGGACAAACGTTATTCGGAATCACTGGGCTTAAAGAGTGCGTAGGCGGTCTACCAAGTTGGGTGTGAAATCCC

TCGGCTCAACCGAGGAATTGCGCTCAAAACTGGCAGACTCGAGGAAGGCAGGGGTAAGCGGAACTGATGGTGGAGCGGTG

AAATGCGTTGATATCATCAGGAACACCAGTGGCGAAGGCGGCTTACTGGGCCTTTTCTGACGCTGAGGCACGAAAGCTAG

GGTAACGAACGGG

>JQ346728.1 protochlamydia_amoebophila_jq346728

TACGGAGGGTGCAAGCATTAATCGGATTTATTGGGCGTAAAGGGCGCGTAGGCGGGGGTGTCAGTCAGATGTGAAATCCC

GAGGCTCAACCTCGGAACAGCATTTGAAACTACATCTCTAGAGGGTAGGCGGAGAAAACGGAATTCCACAAGTAGCGGTG

AAATGCGTAGATATGTGGAAGAACACCGGTGGCGAAGGCGGTTTTCTAGCTTATACCTGACGCTAAAGCGCGAAAGCAAG

GGGATCAAACAGG

>JQ346773.1 spirochaetes_jq346773

CACGTAGGGGGCGAGCGTTGTTCGGAATTACTGGGCGTAAAGGGCATGTAGGCGGCTTGGTAAGCCTGGCGTGAAAGTCT

GCAGCTTAACTGTAGGGATGCGTTGGGAACTGCTTAGCTTGAGTTACGGAGAGGGAGCTGGAATTCCTGGTGTAGGGGTG

AAATCTGTAGATATCAGGAAGAACACCGATGGCGAAGGCAAGCTTCTGGCCGACAACTGACGCTGAGGTGCGAAAGTGTG

GGGATCAAACAGG

>JQ346738.1 sulfurihydrogenibium_yellowstonense_jq346738

TACGTAGGTCCCGAACGTTGCGCGAATTTACTGGGCGTAAAGGGTCCGTAGGCGGTTTAGCAAGTGGTTGGTGAAATTTC

ACGGCTCAACCGTGAAACTGCCTTCCAAACTGCTAAACTTGAGGCAGGGAGAGGTCGGCGGAATTCCCGGTGTAGCGGTG

AAATGCGTAGATATCGGGAGGAACACCAGTGGCGAAGGCGGCCGACTGGAACTGTCCTGACGCTGAGGGACGAAAGCTAG

GGGAGCAAACCGG

>JQ346774.1 synergistetes_jq346774

TACGTAGGGGGCAAGCGTTGTCCGGAATTACTGGGCGTAAAGCGCACGCAGGCTGAATTATAAGTCGGTAGTCAAAGGCG

GAGGCTCAACCTCTGTATATCTATCGATACTGTAAATCTGGAGTATGTGAGAGGGAAGCGGAATTCCCGGTGTAGCGGTG

AAATGCGTAGATATCGGGAGGAACACCAGTGGCGAAGGCGGCTTCCTGGCACAATACTGACGCTCATGTGCGAAAGCTAG

GGCAGCGAACGGG

>JQ346744.1 syntrophobacter_fumaroxidans_jq346744

TACGGAGGGTGCGAGCGTTATTCGGAATTACTGGGCGTAAAGCGCGTGCAGGCGGTTTGGCAAGTCTGATGTGAAAGCCC

CGGGCTTAACCTGGGAAGTGCATTGGAAACTGCCGGACTTGAGTACTGGAGAGGAAGGGGGAATTCCCGGTGTAGAGGTG

AAATTCGTAGAGATCGGGAGGAATACCAGTGGCGAAGGCGCCCTTCTGGACGGTTACTGACGCTGAGACGCGAAAGCGTG

GGGAGCAAACAGG

>JQ346731.1 syntrophococcus_sucromutans_jq346731

TACGTAGGGGGCAAGCGTTATCCGGATTTACTGGGTGTAAAGGGAGCGCAGACGGCAGTGCAAGTCTGAAGTGAAAACCC

GAGGCTCAACCACGGGATTGCTTTGGAAACTGTACAGCTAGAGTGTCGGAGGGGTAAGCGGAATTCCTAGTGTAGCGGTG

AAATGCGTAGATATTAGGAGGAACACCGGTGGCGAAGGCGGCTTACTGGACGATAACTGACGTTGAGGCTCGAAAGCGTG

GGGAGCAAACAGG

>JQ346743.1 syntrophothermus_lipocalidus_jq346743

GACGTAGGGGGCGAGCGTTGTCCGGAATTACTGGGCGTAAAGAGCGTGTAGGCGGGCTGTTAAGTCAGGTGTGAAATACC

GCAGCTCAACTGCGGGGTGGCATCTGATACTGGCAGTCTTGAGGGCAGGAGAGGGAAGTGGAATTCCTGGTGTAGCGGTG

AAATGCGTAGATATCAGGAGGAACACCAGTGGCGAAGGCGGCTTCCTGGACTGGCCCTGACGCTGAGACGCGAAAGCGTG

GGGAGCAAACAGG

>JQ346736.1 syntrophus_buswellii_jq346736

TACGGGGGGTGCTAGCGTTGTTCGGAATCATTGGGCGTAAAGAGCGTGTAGGCGGCTAGGCAAGTCAGATGTGAAATCCC

TGGGCTTAACCCAGGACGTGCATTTGAAACTGCTTGGCTTGAGTAGGGAAGAGGGAAGTGGTATTCCTGGTGTAGAGGTG

AAATTCGTAGATATCAGGAGGAACACCGGTGGCGAAGGCGACTTCCTGGTCCTATACTGACGCTGAGACGCGAGAGCGTG

GGGAGCAAACAGG

>JQ346737.1 syntrophus_gentianae_jq346737

TACGGGGGGTGCAAGCGTTGTTCGGAATCATTGGGCGTAAAGAGCGTGTAGGCGGCTAGGCAAGTCAGATGTGAAATCCC

TGGGCTTAACCCAGGACGTGCATTTGAAACTGCTTGGCTTGAGTAAGGAAGAGGGAAGTGGAATTCCTGGTGTAGAGGTG

AAATTCGTAGATATCAGGAGGAACACCGGTGGCGAAGGCGACTTCCTGGTCCTATACTGACGCTGAGACGCGAGAGCGTG

GGGAGCAAACAGG

>JQ346745.1 thermodesulfobacterium_commune_jq346745

TACGCAGGTGGCGAGCGTTGCCCGGAATTACTGGGCGTAAAGGGTGCGTAGGCGGCCGGACAAGTCATAGGTTAAAGCCC

GGAGCTCAACTCCGGAAAGGCCTATGATACTGTCTGGCTTGAGGGCCGGAGAGGCTGGCGGAATTCCCGGTGTAGGGGTG

AAATCCGTAGATATCGGGAGGAACACCGGTGGGGAAGCCGGCCAGCTGGACGGTTCCTGACGCTGAGGCACGAAAGCGTG

GGGAGCAAACCGG

>JQ346739.1 thermomicrobium_roseum_jq346739

GACGTAGGGGGCGAGCGTTACCCGGAGTCACTGGGCGTAAAGGGCGTGTAGGCGGCTGGGTACGCCGCGTGTGAAAGTCC

CCGGCTCAACCGGGGAGGGTCGCGCGGGACGGCCTGGCTCGAGGGCGGGAGAGGCGGGTGGAATTCCCGGTGTAGCGGTG

AAATGCGTAGAGATCGGGAGGAACGCCGGTGGCGAAGGCGGCCCGCTGGCCCGTACCTGACGCTGAGGCGCGAAGGCGTG

GGGAGCGAACCGG

>JQ346746.1 thermotoga_neapolitana_jq346746

TACGTAGGGGGCAAGCGTTACCCGGATTTACTGGGCGTAAAGGGGGCGTAGGCGGCCTGGTGTGTCGGACGTGAAATCCC

ACGGCTCAACCGTGGGGCTGCATCCGAAACTACCAGGCTTGGGGGCGGTAGAGGGAGACGGAACTGCCGGTGTAGGGGTG

AAATCCGTAGATATCGGCAGGAACGCCGGTGGGGAAGCCGGTCTCCTGGGCCGACCCCGACGCTGAGGCCCGAAAGCCAG

GGGAGCAAACCGG

>JQ346775.1 verrucomicrobia_jq346775

TACGAAGGTCCCGAGCGTTGTTCGGAATCACTGGGCGTAAAGGGAGCGTAGGCGGCGTGGTAAGTCAGATGTGAAATCCC

GGGGCTCAACCCCGGAACTGCATCCGATACTGCCGTGCTAGAGGAATGGAGAGGTAGCTGGAATTCTTGGTGTAGCAGTG

AAATGCGTGGATATCAAGAGGAACACTCGTGGCGAAAGCGAGCTACTGGACATTTTCTGACGCTGAGGCTCGAAGGCTAG

GGTAGCGAAAGGG

>JQ346729.1 victivallis_vadensis_jq346729

TACGTAGGTGGCGAGCGTTGTTCGGATTTATTGGGCGTAAAGGGTCTGTAGGAGGTTTGTTAAATACGAGGTGAAATCCG

GGGGCTCAACTTCCGAATTGCCTTGTAGACTGATGAACTAGAGTACTGGAGAGGTAAGCGGAATACCAGGTGTAGCGGTG

GAATGCGTAGATATCTGGTAGAACACCAATAGCGAAGGCAGCTTGCTGGACAGAAACTGACTCTGAAAGACGAAAGCATG

GGGAGCAAACAGG
