# Supplementary material for: Effects of error, chimera, bias, and GC content on the accuracy of amplicon sequencing
Source: mSystems. 2023 Dec 1;8(6):e01025-23. doi: 10.1128/msystems.01025-23 (PMC10734440; doi:10.1128/msystems.01025-23)
Supplement: Supplemental figures — Figures S1-S4. [file msystems.01025-23-s0002.docx]

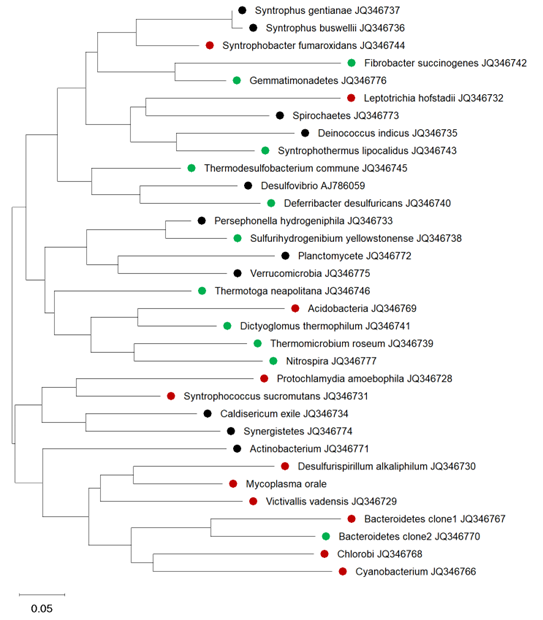


**Fig. S1.** **The phylogenetic tree to visualize the similarities between the 33 mock community strains.** Red dots depict the low GC content strains and green dots depict the high GC content strains.


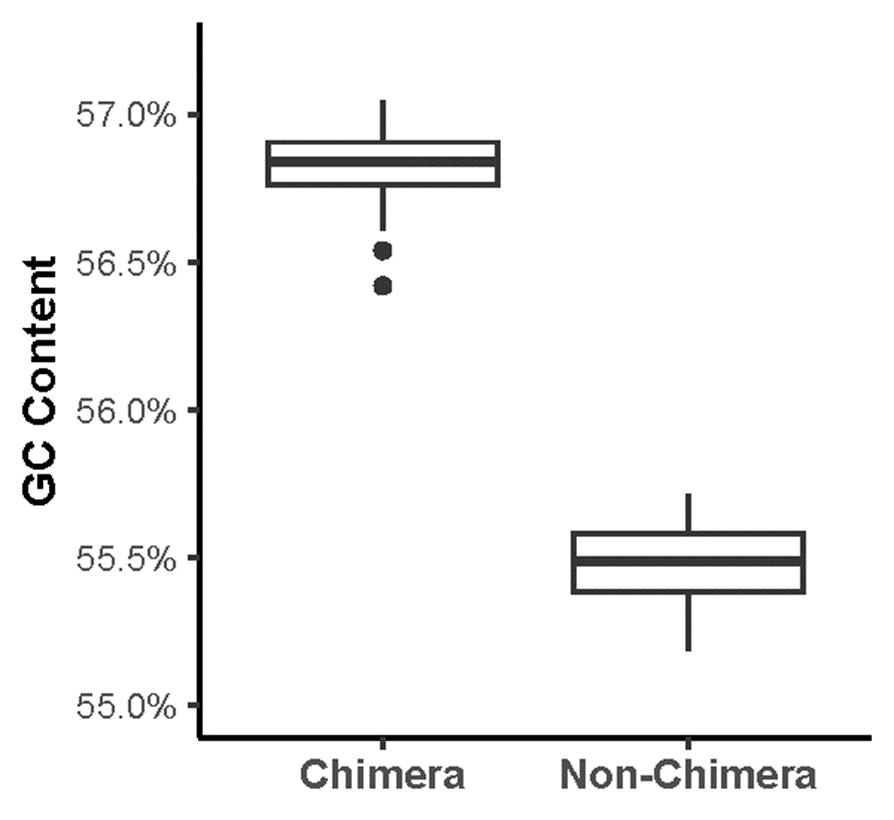


**Fig. S2. The box plot illustrating the GC contents of the chimeric and non-chimeric reads in the Bm1 trimmed (Q20-W2) community.** Boxes and whiskers indicate quartiles and black line indicate mean values. Two tail T Test was done to compare GC contents between the chimeric and non-chimeric reads which showed the significant difference (p<0.001).


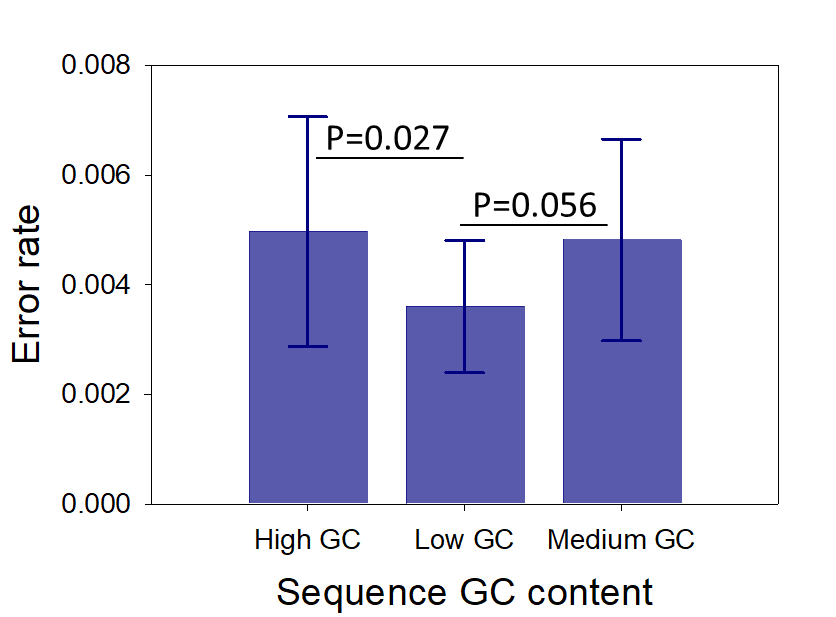


**Fig. S3.** **Error rates of the high, medium and low GC content strain clusters of Bm1.** Error rates were calculated for the high, medium, and low GC content strain clusters of Bm1. An ANOVA was conducted to compare the mean errors of the three clusters, followed by Tukey's multiple comparisons test. The resulting p-values are reported for each comparison.


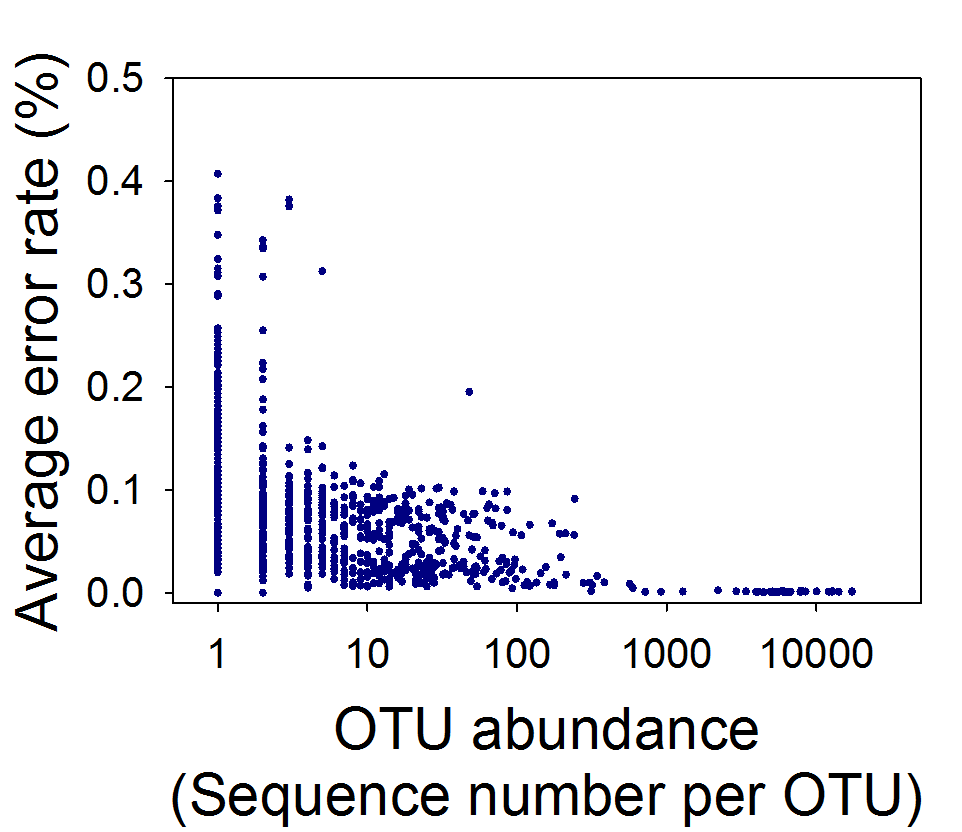


**Fig. S4. Relationship between error rate and OTU abundance.** Average error rate of each OTU across all libraries, including libraries for all three mock communities and with all three library generation methods and their replicates, was calculated and plotted against the OTU abundance (the sequence number of each OTU).
