# Supplementary material for: Effects of error, chimera, bias, and GC content on the accuracy of amplicon sequencing
Source: mSystems. 2023 Dec 1;8(6):e01025-23. doi: 10.1128/msystems.01025-23 (PMC10734440; doi:10.1128/msystems.01025-23)
Supplement: Supplemental materials — List of the supplemental files and legends. [file msystems.01025-23-s0003.docx]

**Supplemental materials**

**Figure S1.** The phylogenetic tree to visualize the similarities between the 33 mock community strains. Red dots depict the low GC content strains and green dots depict the high GC content strains.

**Figure S2.** The box plot illustrating the GC contents of the chimeric and non-chimeric reads in the Bm1 trimmed (Q20-W2) community. Boxes and whiskers indicate quartiles and black line indicate mean values. Two tail T Test was done to compare GC contents between the chimeric and non-chimeric reads which showed the significant difference (p<0.001).

**Figure S3.** Error rates of the high, medium and low GC content strain clusters of Bm1. Error rates were calculated for the high, medium, and low GC content strain clusters of Bm1. An ANOVA was conducted to compare the mean errors of the three clusters, followed by Tukey's multiple comparisons test. The resulting p-values are reported for each comparison.

**Figure S4.** Relationship between error rate and OTU abundance. Average error rate of each OTU across all libraries, including libraries for all three mock communities and with all three library generation methods and their replicates, was calculated and plotted against the OTU abundance (the sequence number of each OTU).

**Table S1.** Summary of Sequence data statistics (raw data).

**Table S2.** (A) Number of chimeras in raw sequence reads (Bm1), (B) Number of chimeras in raw sequence reads (Bm2), and (C) Number of chimeras in raw sequence reads (Bm3).

**Table S3.** (A) Error rate of raw sequences and sequences after processing and methods comparisons-Bm1, (B) Error rate of raw sequences and sequences after processing and methods comparisons-Bm3, and (C) Error rate of raw sequences and sequences after processing and methods comparisons-Bm2.

**Table S4.** OTU/sequence detection by UPARSE and artifacts sources analysis when a balanced mode was used for chimera identification.

**Table S5**. Sources of artifacts when zOTUs were detected and classed by UPARSE and a sensitive mode was used for chimera identification.

**Table S6**. (A) Methods comparisons in OTU or ASV detection and artifact composition and sources when a balanced mode was used for chimera detection, and (B) Methods comparisons in artifact composition and sources when a sensitive mode was used for chimera identification.

**Table S7.** Contaminants (ASVs or (z)OUTs) detected from Bm3 using different data processing methods.

**Table S8.** (A) Sources of the spurious sequences-Bm1, (B) Sources of the spurious sequences-Bm2, (C) Sources of the spurious sequences-Bm3.

**Table S9**. (A) Mock community strains observed ratio and differences to expected- Bm1, (B) Mock community strains observed ratio and differences to expected- Bm2, (C) Mock community strains observed ratio and differences to expected- Bm3.

**Table S10.** (A) Statistics of Template Free control 16S Amplicon Sequence – Lab Contaminants, (B) Statistics of Template Free control 16S Amplicon Sequence – Lab Contaminants -Phyla, (C) Statistics of Template Free control 16S Amplicon Sequence – Lab Contaminants -Genera.

**Table S11.** Mock community strains.

**Table S12.** (A) Forward and sequencing primers for non-phasing method, and (B) Assignment of non-phasing reverse primers for tagged PCR libraries with non-phasing method. (C) Phasing Forward Primers, (D) Assignment of phasing reverse primers for tagged PCR libraries with one-step phasing method, (E) Assignment of phasing reverse primers for tagged PCR libraries with two-step phasing method.

**File S1.** Bacteria mock community strain V4 sequences.
