# Supplementary material for: Effects of error, chimera, bias, and GC content on the accuracy of amplicon sequencing
Source: mSystems. 2023 Dec 1;8(6):e01025-23. doi: 10.1128/msystems.01025-23 (PMC10734440; doi:10.1128/msystems.01025-23)
Supplement: Supplemental tables, set 1 — Tables S1-S8 and S11. [file msystems.01025-23-s0004.docx]

| **Table S1. Summary of Sequence data statistics (raw data) ^a^** | | | | | | | | | |
| --- | --- | --- | --- | --- | --- | --- | --- | --- | --- |
| **Replicate** | **Non-phasing** | | | **One-step phasing** | | | **Two-step phasing** | | |
|  | **Bm1** | **Bm2** | **Bm3** | **Bm1** | **Bm2** | **Bm3** | **Bm1** | **Bm2** | **Bm3** |
| 1 | 28002 | 36012 | 26496 | 10330 | 10206 | 12289 | 30569 | 20629 | 19742 |
| 2 | 23763 | 28318 | 23410 | 6638 | 9326 | 13190 | 21662 | 10682 | 15735 |
| 3 | 33906 | 20237 | 20869 | 18631 | 11559 | 11163 | 32654 | 17315 | 35515 |
| 4 | 27536 | 16240 | 15544 | 9904 | 12894 | 10776 | 15867 | 15953 | 27762 |
| 5 | 24901 | 24673 | 18918 | 10161 | 8094 | 10256 | 21647 | 25624 | 16179 |
| 6 | 25188 | 14807 | 21454 | 11358 | 9338 | 8442 | 20201 | 16778 | 17897 |
| 7 | 27553 | 25492 | 28876 | 12670 | 8015 | 6773 | 16281 | 12648 | 12306 |
| 8 | 22869 | 20132 | 25560 | 7917 | 9184 | 8294 | 8840 | 12903 | 8400 |
| 9 | 21621 | 25906 | 31831 | 12221 | 11242 | 9352 | 26210 | 25149 | 26014 |
| 10 | 23762 | 27989 | 27064 | 10348 | 11861 | 10681 | 23310 | 17430 | 14921 |
| 11 | 28671 | 25692 | 25909 | 9272 | 8894 | 6536 | 13901 | 10512 | 14751 |
| 12 | 25838 | 25214 | 28028 | 7657 | 8425 | 7131 | 17403 | 8394 | 14972 |
| 13 | 22989 | 26184 | 26015 | 7667 | 9087 | 9187 | 14264 | 14150 | 25195 |
| 14 | 21364 | 22301 | 26779 | 11919 | 18898 | 10635 | 29146 | 34956 | 20886 |
| 15 | 20717 | 17264 | 31607 | 8042 | 10731 | 12707 | 12128 | 10210 | 22406 |
| 16 | 21223 | 30577 | 24715 | 7135 | 19557 | 8595 | 14456 | 33765 | 13273 |
| 17 | 17500 | 19240 | 29594 | 8568 | 10286 | 10651 | 16515 | 28255 | 23569 |
| 18 | 27912 | 29086 | 30330 | 14148 | 19743 | 8715 | 12849 | 26441 | 14647 |
| 19 | 26410 | 29865 | 31667 | 7572 | 15923 | 11069 | 15938 | 38880 | 21480 |
| 20 | 27774 | 31591 | 28867 | 8175 | 12498 | 14312 | 11772 | 23310 | 20661 |
| 21 | 34166 | 22947 | 26315 | 6256 | 5688 | 7725 | 11863 | 11322 | 19906 |
| 22 | 20462 | 26391 | 27404 | 11852 | 8148 | 10516 | 20757 | 16715 | 20538 |
| 23 | 25993 | 25546 | 17098 | 8984 | 8128 | 8214 | 12041 | 19015 | 14138 |
| 24 | 23503 | 15439 | 22716 | 9122 | 12438 | 10628 | 11169 | 11156 | 11797 |

^a^ Number in the table are paired reads.

| **Table S2A. Number of chimeras in raw sequence reads (Bm1) ^a^** | | | | | | | | | | | |
| --- | --- | --- | --- | --- | --- | --- | --- | --- | --- | --- | --- |
|  |  | Total sequence ^b^ | Total | | | Detected | | | Un-detected | | |
|  |  |  | Chimera ^c^ | % | Significance ^d^ | Chimera ^c^ | % | Significance ^d^ | Chimera ^c^ | % | Significance ^d^ |
| Forward reads | Non-phasing | 195190 | 812 (143) | 10.01 (1.03) | a | 525 (95) | 6.47 (0.75) | a | 287 (53) | 3.54 (0.38) | a |
|  | One-step phasing | 217835 | 909 (361) | 10.03 (3.25) | a | 564 (219) | 6.23 (2.01) | a | 346 (146) | 3.80 (1.31) | a |
|  | Two-step phasing | 217729 | 520 (329) | 5.83 (3.00) | b | 339 (207) | 3.81 (1.93) | b | 181 (123) | 2.01 (1.10) | b |
| Reverse reads | Non-phasing | 130473 | 512(91) | 9.44 (1.06) | a | 388 (68) | 7.18 (0.90) | a | 124 (27) | 2.27 (0.29) | a |
|  | One-step phasing | 212824 | 897 (385) | 10.10 (3.41) | a | 697 (302) | 7.86 (2.72) | a | 200 (87) | 2.24 (0.75) | a |
|  | Two-step phasing | 219924 | 496 (327) | 5.51 (2.89) | b | 396 (260) | 4.40 (2.30) | b | 100(69) | 1.11 (0.61) | b |
| Joined Sequences | Non-phasing | 235870 | 1038 (181) | 10.59 (1.13) | a | 720 (133) | 7.35 (0.89) | a | 318 (56) | 3.25 (0.37) | a |
|  | One-step phasing | 241683 | 1149 (472) | 11.40 (3.69) | a | 822 (339) | 8.15 (2.66) | a | 327 (137) | 3.24 (1.08) | a |
|  | Two-step phasing | 238884 | 641 (425) | 6.54 (3.42) | b | 469 (307) | 4.79 (2.47) | b | 172 (119) | 1.75 (0.96) | b |

^a^ Per mock community per method involved 24 replicates. Data in parenthesis is standard deviation.

^b^ The sum of all 24 replicates.

^c^ The average of the 24 replicates.

^d^ Lowercase letters (i.e., a, b, ab, and c) show the results of ANOVA and LSD tests to examine the significant differences.

| **Table S2B. Number of chimeras in raw sequence reads (Bm2) ^a^** | | | | | | | | | | | |
| --- | --- | --- | --- | --- | --- | --- | --- | --- | --- | --- | --- |
|  |  | Total sequences ^b^ | Total | | | Detected | | | Un-detected | | |
|  |  |  | Chimeras ^c^ | % | Significance ^d^ | Chimeras ^c^ | % | Significance ^d^ | Chimeras ^c^ | % | Significance ^d^ |
| Forward reads | Non-phasing | 211280 | 251 (63) | 2.87 (0.44) | a | 164 (43) | 1.87 (0.31) | b | 87 (23) | 1.00 (0.19) | a |
|  | One-step phasing | 229496 | 273 (91) | 2.93 (0.77) | a | 208 (76) | 2.24 (0.68) | a | 65 (23) | 0.69 (0.19) | b |
|  | Two-step phasing | 224212 | 169 (81) | 1.84 (0.65) | b | 131 (61) | 1.44 (0.48) | c | 38 (21) | 0.41 (0.19) | c |
| Reverse reads | Non-phasing | 143086 | 146 (45) | 2.44 (0.38) | a | 111 (35) | 1.87 (0.33) | b | 34 (12) | 0.57 (0.11) | a |
|  | One-step phasing | 224154 | 245 (88) | 2.70 (0.76) | a | 207 (78) | 2.29 (0.69) | a | 38 (16) | 0.42 (0.14) | b |
|  | Two-step phasing | 224279 | 150 (74) | 1.62 (0.56) | b | 130 (64) | 1.41 (0.48) | c | 20 (12) | 0.21 (0.11) | c |
| Joined Sequences | Non-phasing | 238870 | 292 (69) | 2.95 (0.35) | a | 198 (49) | 1.99 (0.26) | b | 95 (23) | 0.96 (0.15) | a |
|  | One-step phasing | 242882 | 313 (105) | 3.19 (0.85) | a | 244 (88) | 2.50 (0.77) | a | 69 (26) | 0.69 (0.19) | b |
|  | Two-step phasing | 240258 | 192 (93) | 1.94 (0.64) | b | 154 (73) | 1.56 (0.51) | c | 38 (21) | 0.38 (0.16) | c |

^a^ Per mock community per method involved 24 replicates. Data in parenthesis is standard deviation.

^b^ The sum of all 24 replicates.

^c^ The average of the 24 replicates.

^d^ Lowercase letters (i.e., a, b, ab, and c) show the results of ANOVA and LSD tests to examine the significant differences.

| **Table S2C. Number of chimeras in raw sequence reads (Bm3) ^a^** | | | | | | | | | | | |
| --- | --- | --- | --- | --- | --- | --- | --- | --- | --- | --- | --- |
|  |  | Total sequences ^b^ | Total | | | Detected | | | Un-detected | | |
|  |  |  | Chimeras ^c^ | % | Significance ^d^ | Chimeras ^c^ | % | Significance ^d^ | Chimeras ^c^ | % | Significance ^d^ |
| Forward reads | Non-phasing | 166949 | 637 (124) | 9.20 (1.20) | a | 451 (84) | 6.53 (0.83) | a | 185 (43) | 2.67 (0.45) | a |
|  | One-step phasing | 197239 | 780 (302) | 9.40 (2.86) | a | 565 (228) | 6.79 (2.11) | a | 215 (84) | 2.60 (0.88) | a |
|  | Two-step phasing | 203632 | 399 (212) | 4.52 (1.64) | b | 281 (150) | 3.18 (1.14) | b | 119 (63) | 1.35 (0.51) | b |
| Reverse reads | Non-phasing | 104422 | 410 (88) | 9.47 (1.19) | a | 303 (62) | 7.00 (0.86) | b | 107 (29) | 2.47 (0.48) | a |
|  | One-step phasing | 193088 | 832 (343) | 10.16 (3.17) | a | 671 (286) | 8.19 (2.62) | a | 161 (65) | 1.98 (0.66) | b |
|  | Two-step phasing | 211905 | 414 (229) | 4.48 (1.68) | b | 332 (187) | 3.58 (1.37) | c | 82 (44) | 0.90 (0.34) | c |
| Joint Sequences | Non-phasing | 231778 | 952 (180) | 9.89 (1.15) | b | 694 (130) | 7.21 (0.86) | b | 258 (54) | 2.68 (0.38) | a |
|  | One-step phasing | 240312 | 1144 (454) | 11.26 (3.41) | a | 894 (364) | 8.79 (2.70) | a | 250 (97) | 2.47 (0.80) | a |
|  | Two-step phasing | 236852 | 537 (289) | 5.19 (1.88) | c | 409 (221) | 3.96 (1.45) | c | 128 (70) | 1.23 (0.47) | b |

^a^ Per mock community per method involved 24 replicates. Data in parenthesis is standard deviation.

^b^ The sum of all 24 replicates.

^c^ The average of the 24 replicates.

^d^ Lowercase letters (i.e., a, b, ab, and c) show the results of ANOVA and LSD tests to examine the significant differences.

| **Table S3A. Error rate of raw sequences and sequences after processing and methods comparisons-Bm1**^a^ | | | | | | | | | | | |
| --- | --- | --- | --- | --- | --- | --- | --- | --- | --- | --- | --- |
|  | | | Forward Reads | | | Reverse Reads | | | Joined sequences | | |
|  | | | non-phasing ^c^ | One-step phasing | Two-step phasing | non-phasing | One-step phasing | Two-step phasing | non-phasing | One-step phasing | Two-step phasing |
| Raw | w/ chimera | rate | 1.30% | 1.13% | 0.91% | 1.63% | 0.98% | 0.83% | 0.84% | 0.86% | 0.65% |
|  |  | stdv | 0.09% | 0.17% | 0.16% | 0.13% | 0.24% | 0.22% | 0.08% | 0.24% | 0.22% |
|  |  | Significance ^b^ | a | b | c | a | b | c | a | a | b |
|  | w/o chimera | rate | 0.96% | 0.84% | 0.72% | 1.24% | 0.50% | 0.56% | 0.44% | 0.42% | 0.39% |
|  |  | stdv | 0.05% | 0.09% | 0.09% | 0.12% | 0.09% | 0.09% | 0.04% | 0.10% | 0.09% |
|  |  | Significance ^b^ | a | b | c | a | b | b | a | ab | b |
| Trim Q20-W5 | w/ chimera | rate | 0.78% | 0.69% | 0.53% | 0.85% | 0.85% | 0.59% | 0.79% | 0.84% | 0.59% |
|  |  | stdv | 0.07% | 0.19% | 0.17% | 0.08% | 0.25% | 0.21% | 0.08% | 0.24% | 0.22% |
|  |  | Significance ^b^ | a | a | b | a | a | b | a | a | b |
|  | w/o chimera | rate | 0.43% | 0.38% | 0.34% | 0.41% | 0.36% | 0.31% | 0.40% | 0.40% | 0.33% |
|  |  | stdv | 0.03% | 0.09% | 0.08% | 0.03% | 0.09% | 0.07% | 0.04% | 0.10% | 0.09% |
|  |  | Significance ^b^ | a | b | b | a | b | c | a | a | b |
| Trim Q20-W2 | w/ chimera | rate | 0.74% | 0.67% | 0.50% | 0.80% | 0.81% | 0.55% | 0.78% | 0.83% | 0.56% |
|  |  | stdv | 0.08% | 0.19% | 0.16% | 0.09% | 0.24% | 0.21% | 0.08% | 0.24% | 0.21% |
|  |  | Significance | a | a | b | a | a | b | a | a | b |
|  | w/o chimera | rate | 0.42% | 0.38% | 0.31% | 0.35% | 0.33% | 0.27% | 0.39% | 0.40% | 0.30% |
|  |  | stdv | 0.04% | 0.10% | 0.08% | 0.03% | 0.08% | 0.07% | 0.04% | 0.10% | 0.08% |
|  |  | Significance ^b^ | a | a | b | a | a | b | a | a | b |
| Trim Q25-W5 | w/ chimera | ratio | 0.75% | 0.67% | 0.51% | 0.81% | 0.83% | 0.56% | 0.78% | 0.83% | 0.57% |
|  |  | stdv | 0.07% | 0.19% | 0.17% | 0.08% | 0.25% | 0.21% | 0.08% | 0.24% | 0.22% |
|  |  | Significance ^b^ | a | a | b | a | a | b | a | a | b |
|  | w/o chimera | rate | 0.42% | 0.38% | 0.32% | 0.35% | 0.34% | 0.28% | 0.39% | 0.40% | 0.31% |
|  |  | stdv | 0.03% | 0.09% | 0.08% | 0.03% | 0.09% | 0.07% | 0.04% | 0.10% | 0.09% |
|  |  | Significance ^b^ | a | a | b | a | a | b | a | a | b |
| Trim Q25-W2 | w/ chimera | rate | 0.69% | 0.63% | 0.47% | 0.75% | 0.80% | 0.51% | 0.77% | 0.80% | 0.55% |
|  |  | stdv | 0.08% | 0.18% | 0.16% | 0.10% | 0.24% | 0.20% | 0.08% | 0.23% | 0.21% |
|  |  | Significance ^b^ | a | a | b | a | a | b | a | a | b |
|  | w/o chimera | rate | 0.39% | 0.37% | 0.29% | 0.29% | 0.31% | 0.24% | 0.39% | 0.38% | 0.29% |
|  |  | stdv | 0.04% | 0.09% | 0.08% | 0.04% | 0.08% | 0.06% | 0.04% | 0.10% | 0.08% |
|  |  | Significance ^b^ | a | a | b | a | a | b | a | a | b |
| Trim Q30-W5 | w/ chimera | rate | 0.70% | 0.63% | 0.49% | 0.78% | 0.80% | 0.52% | 0.77% | 0.82% | 0.55% |
|  |  | stdv | 0.07% | 0.18% | 0.17% | 0.11% | 0.24% | 0.20% | 0.09% | 0.23% | 0.21% |
|  |  | Significance ^b^ | a | a | b | a | a | b | a | a | b |
|  | w/o chimera | rate | 0.40% | 0.36% | 0.30% | 0.31% | 0.31% | 0.25% | 0.38% | 0.39% | 0.30% |
|  |  | stdv | 0.04% | 0.09% | 0.08% | 0.05% | 0.08% | 0.07% | 0.04% | 0.10% | 0.09% |
|  |  | Significance ^b^ | a | a | b | a | a | b | a | a | b |
| Trim Q30-W2 | w/ chimera | rate | 0.64% | 0.58% | 0.46% | 0.75% | 0.76% | 0.49% | 0.75% | 0.80% | 0.52% |
|  |  | stdv | 0.08% | 0.17% | 0.16% | 0.17% | 0.23% | 0.21% | 0.09% | 0.23% | 0.21% |
|  |  | Significance ^b^ | a | a | b | a | a | b | a | a | b |
|  | w/o chimera | rate | 0.39% | 0.36% | 0.28% | 0.25% | 0.27% | 0.21% | 0.39% | 0.38% | 0.27% |
|  |  | stdv | 0.06% | 0.10% | 0.08% | 0.07% | 0.08% | 0.06% | 0.04% | 0.10% | 0.08% |
|  |  | Significance ^b^ | a | a | b | ab | a | b | a | a | b |

^a^ w/ chimera, with chimera, a chimera removal step was not used; w/o chimera, without chimera, chimera detected by UCHIME using the greengenes database were removed.
^b^ Lowercase letters (i.e., a, b, ab, and c) show the results of ANOVA and LSD tests to examine the significant differences.

^c^ Per method involved 24 replicates.

| **Table S3B. Error rate of raw sequences and sequences after processing and methods comparisons-Bm3** ^a^ | | | | | | | | | | | |
| --- | --- | --- | --- | --- | --- | --- | --- | --- | --- | --- | --- |
|  | | | Forward Reads | | | Reverse Reads | | | Joined sequences | | |
|  | | | non-phasing ^c^ | One-step phasing | Two-step phasing | non-phasing | One-step phasing | Two-step phasing | non-phasing | One-step phasing | Two-step phasing |
| Raw | w/ chimera | rate | 1.70% | 1.62% | 1.14% | 1.93% | 0.99% | 0.79% | 0.77% | 0.79% | 0.55% |
|  |  | stdv | 0.11% | 0.17% | 0.10% | 0.16% | 0.19% | 0.11% | 0.07% | 0.20% | 0.12% |
|  |  | Significance ^b^ | a | b | c | a | b | c | a | a | b |
|  | w/o chimera | rate | 1.42% | 1.37% | 1.01% | 1.58% | 0.49% | 0.57% | 0.39% | 0.33% | 0.34% |
|  |  | stdv | 0.10% | 0.13% | 0.06% | 0.15% | 0.06% | 0.06% | 0.03% | 0.07% | 0.05% |
|  |  | Significance ^b^ | a | a | b | a | a | b | a | c | b |
| Trim Q20-W5 | w/ chimera | rate | 0.70% | 0.62% | 0.44% | 0.79% | 0.80% | 0.49% | 0.70% | 0.77% | 0.47% |
|  |  | stdv | 0.07% | 0.16% | 0.10% | 0.08% | 0.21% | 0.11% | 0.07% | 0.20% | 0.12% |
|  |  | Significance ^b^ | a | b | c | a | a | b | a | a | b |
|  | w/o chimera | rate | 0.37% | 0.30% | 0.29% | 0.38% | 0.30% | 0.26% | 0.33% | 0.31% | 0.26% |
|  |  | stdv | 0.03% | 0.07% | 0.05% | 0.03% | 0.06% | 0.04% | 0.03% | 0.07% | 0.05% |
|  |  | Significance ^b^ | a | b | b | a | b | c | a | a | b |
| Trim Q20-W2 | w/ chimera | rate | 0.70% | 0.59% | 0.42% | 0.75% | 0.76% | 0.45% | 0.69% | 0.75% | 0.44% |
|  |  | stdv | 0.08% | 0.16% | 0.10% | 0.11% | 0.21% | 0.11% | 0.07% | 0.20% | 0.12% |
|  |  | Significance ^b^ | a | b | c | a | a | b | a | a | b |
|  | w/o chimera | rate | 0.37% | 0.29% | 0.26% | 0.31% | 0.27% | 0.22% | 0.32% | 0.30% | 0.24% |
|  |  | stdv | 0.04% | 0.07% | 0.05% | 0.05% | 0.06% | 0.04% | 0.03% | 0.07% | 0.05% |
|  |  | Significance ^b^ | a | b | b | a | b | c | a | a | b |
| Trim Q25-W5 | w/ chimera | ratio | 0.69% | 0.59% | 0.42% | 0.77% | 0.77% | 0.46% | 0.69% | 0.76% | 0.45% |
|  |  | stdv | 0.08% | 0.16% | 0.10% | 0.10% | 0.21% | 0.11% | 0.07% | 0.20% | 0.12% |
|  |  | Significance ^b^ | a | b | c | a | a | b | a | a | b |
|  | w/o chimera | rate | 0.36% | 0.28% | 0.27% | 0.32% | 0.28% | 0.23% | 0.32% | 0.30% | 0.24% |
|  |  | stdv | 0.04% | 0.07% | 0.05% | 0.04% | 0.06% | 0.04% | 0.03% | 0.07% | 0.05% |
|  |  | Significance ^b^ | a | b | b | a | b | c | a | a | b |
| Trim Q25-W2 | w/ chimera | rate | 0.68% | 0.55% | 0.40% | 0.72% | 0.71% | 0.41% | 0.69% | 0.73% | 0.43% |
|  |  | stdv | 0.09% | 0.15% | 0.10% | 0.15% | 0.20% | 0.12% | 0.07% | 0.20% | 0.12% |
|  |  | Significance ^b^ | a | b | c | a | a | b | a | a | b |
|  | w/o chimera | rate | 0.35% | 0.28% | 0.25% | 0.25% | 0.24% | 0.19% | 0.32% | 0.29% | 0.22% |
|  |  | stdv | 0.04% | 0.07% | 0.05% | 0.05% | 0.05% | 0.04% | 0.03% | 0.07% | 0.05% |
|  |  | Significance ^b^ | a | b | b | a | a | b | a | b | c |
| Trim Q30-W5 | w/ chimera | rate | 0.68% | 0.56% | 0.41% | 0.74% | 0.73% | 0.43% | 0.70% | 0.74% | 0.43% |
|  |  | stdv | 0.09% | 0.15% | 0.10% | 0.13% | 0.20% | 0.12% | 0.07% | 0.20% | 0.12% |
|  |  | Significance ^b^ | a | b | c | a | a | b | a | a | b |
|  | w/o chimera | rate | 0.36% | 0.28% | 0.26% | 0.26% | 0.25% | 0.20% | 0.33% | 0.30% | 0.23% |
|  |  | stdv | 0.05% | 0.07% | 0.05% | 0.05% | 0.06% | 0.04% | 0.03% | 0.07% | 0.05% |
|  |  | Significance ^b^ | a | b | b | a | a | b | a | b | c |
| Trim Q30-W2 | w/ chimera | rate | 0.68% | 0.53% | 0.40% | 0.65% | 0.67% | 0.39% | 0.70% | 0.72% | 0.41% |
|  |  | stdv | 0.12% | 0.14% | 0.11% | 0.19% | 0.19% | 0.12% | 0.08% | 0.20% | 0.11% |
|  |  | Significance ^b^ | a | b | c | a | a | b | a | a | b |
|  | w/o chimera | rate | 0.35% | 0.28% | 0.26% | 0.19% | 0.20% | 0.17% | 0.34% | 0.29% | 0.20% |
|  |  | stdv | 0.06% | 0.07% | 0.06% | 0.08% | 0.04% | 0.04% | 0.04% | 0.07% | 0.05% |
|  |  | Significance ^b^ | a | b | b | a | a | a | a | b | c |

^a^ w/ chimera, with chimera, a chimera removal step was not used; w/o chimera, without chimera, chimera detected by UCHIME using the greengenes database were removed.
^b^ Lowercase letters (i.e., a, b, ab, and c) show the results of ANOVA and LSD tests to examine the significant differences.

^c^ Per method involved 24 replicates.

| **Table S3C. Error rate of raw sequences and sequences after processing and methods comparisons-Bm2** ^a^ | | | | | | | | | | | |
| --- | --- | --- | --- | --- | --- | --- | --- | --- | --- | --- | --- |
|  | | | Forward Reads | | | Reverse Reads | | | Joined sequences | | |
|  | | | non-phasing ^c^ | One-step phasing | Two-step phasing | non-phasing | One-step phasing | Two-step phasing | non-phasing | One-step phasing | Two-step phasing |
| Raw | w/ chimera | rate | 0.47% | 0.37% | 0.46% | 0.92% | 0.42% | 0.51% | 0.33% | 0.34% | 0.34% |
|  |  | stdv | 0.03% | 0.06% | 0.06% | 0.12% | 0.06% | 0.07% | 0.03% | 0.05% | 0.05% |
|  |  | Significance ^b^ | a ^b^ | b | a | a | c | b | a | a | a |
|  | w/o chimera | rate | 0.34% | 0.24% | 0.38% | 0.80% | 0.26% | 0.42% | 0.21% | 0.19% | 0.25% |
|  |  | stdv | 0.02% | 0.03% | 0.04% | 0.11% | 0.03% | 0.05% | 0.01% | 0.02% | 0.03% |
|  |  | Significance ^b^ | b | c | a | a | c | b | b | c | a |
| Trim Q20-W5 | w/ chimera | rate | 0.32% | 0.29% | 0.29% | 0.35% | 0.33% | 0.30% | 0.30% | 0.33% | 0.29% |
|  |  | stdv | 0.03% | 0.06% | 0.05% | 0.03% | 0.05% | 0.05% | 0.03% | 0.05% | 0.04% |
|  |  | Significance ^b^ | a | b | ab | a | a | b | b | a | b |
|  | w/o chimera | rate | 0.20% | 0.15% | 0.21% | 0.22% | 0.16% | 0.20% | 0.18% | 0.18% | 0.19% |
|  |  | stdv | 0.02% | 0.02% | 0.02% | 0.01% | 0.02% | 0.02% | 0.01% | 0.02% | 0.02% |
|  |  | Significance ^b^ | a | b | a | a | c | b | b | b | a |
| Trim Q20-W2 | w/ chimera | rate | 0.30% | 0.28% | 0.26% | 0.32% | 0.31% | 0.27% | 0.28% | 0.32% | 0.26% |
|  |  | stdv | 0.03% | 0.06% | 0.05% | 0.04% | 0.05% | 0.05% | 0.03% | 0.06% | 0.04% |
|  |  | Significance ^b^ | a | ab | b | a | a | b | b | a | b |
|  | w/o chimera | rate | 0.19% | 0.15% | 0.18% | 0.18% | 0.15% | 0.16% | 0.16% | 0.17% | 0.17% |
|  |  | stdv | 0.01% | 0.02% | 0.02% | 0.02% | 0.02% | 0.02% | 0.01% | 0.02% | 0.02% |
|  |  | Significance ^b^ | a | c | b | a | c | b | a | a | a |
| Trim Q25-W5 | w/ chimera | ratio | 0.30% | 0.28% | 0.27% | 0.33% | 0.32% | 0.28% | 0.29% | 0.32% | 0.27% |
|  |  | stdv | 0.03% | 0.06% | 0.05% | 0.03% | 0.05% | 0.05% | 0.03% | 0.05% | 0.04% |
|  |  | Significance ^b^ | a | ab | b | a | a | b | b | a | b |
|  | w/o chimera | rate | 0.19% | 0.15% | 0.18% | 0.18% | 0.15% | 0.18% | 0.17% | 0.17% | 0.18% |
|  |  | stdv | 0.02% | 0.02% | 0.02% | 0.02% | 0.02% | 0.02% | 0.01% | 0.02% | 0.02% |
|  |  | Significance ^b^ | a | b | a | a | b | a | a | a | a |
| Trim Q25-W2 | w/ chimera | rate | 0.26% | 0.26% | 0.24% | 0.29% | 0.30% | 0.24% | 0.27% | 0.30% | 0.25% |
|  |  | stdv | 0.03% | 0.05% | 0.05% | 0.05% | 0.06% | 0.05% | 0.03% | 0.06% | 0.04% |
|  |  | Significance ^b^ | a | a | a | a | a | b | b | a | b |
|  | w/o chimera | rate | 0.17% | 0.14% | 0.15% | 0.14% | 0.13% | 0.14% | 0.15% | 0.15% | 0.16% |
|  |  | stdv | 0.02% | 0.02% | 0.02% | 0.03% | 0.02% | 0.02% | 0.01% | 0.02% | 0.02% |
|  |  | Significance ^b^ | a | b | b | a | b | ab | a | a | a |
| Trim Q30-W5 | w/ chimera | rate | 0.28% | 0.27% | 0.25% | 0.31% | 0.31% | 0.25% | 0.28% | 0.31% | 0.26% |
|  |  | stdv | 0.03% | 0.05% | 0.05% | 0.06% | 0.05% | 0.05% | 0.03% | 0.05% | 0.04% |
|  |  | Significance ^b^ | a | ab | b | a | a | b | b | a | b |
|  | w/o chimera | rate | 0.18% | 0.15% | 0.17% | 0.16% | 0.14% | 0.15% | 0.16% | 0.16% | 0.17% |
|  |  | stdv | 0.02% | 0.02% | 0.02% | 0.03% | 0.02% | 0.02% | 0.01% | 0.02% | 0.02% |
|  |  | Significance ^b^ | a | b | a | a | b | b | a | a | a |
| Trim Q30-W2 | w/ chimera | rate | 0.24% | 0.25% | 0.23% | 0.31% | 0.29% | 0.22% | 0.26% | 0.30% | 0.23% |
|  |  | stdv | 0.03% | 0.05% | 0.05% | 0.11% | 0.06% | 0.05% | 0.03% | 0.05% | 0.04% |
|  |  | Significance ^b^ | a | a | a | a | a | b | b | a | c |
|  | w/o chimera | rate | 0.17% | 0.14% | 0.14% | 0.16% | 0.11% | 0.11% | 0.15% | 0.15% | 0.14% |
|  |  | stdv | 0.02% | 0.02% | 0.02% | 0.07% | 0.03% | 0.02% | 0.02% | 0.02% | 0.02% |
|  |  | Significance ^b^ | b | a | c | a | b | b | a | ab | b |

^a^ w/ chimera, with chimera, a chimera removal step was not used; w/o chimera, without chimera, chimera detected by UCHIME using the greengenes database were removed.
^b^ Lowercase letters (i.e., a, b, ab, and c) show the results of ANOVA and LSD tests to examine the significant differences.

^c^ Per method involved 24 replicates.

| **Table S4.** **OTU/sequence detection by UPARSE and artifacts sources analysis when a balanced mode was used for chimera identification ^a^** | | | | | | | | | | | | | |
| --- | --- | --- | --- | --- | --- | --- | --- | --- | --- | --- | --- | --- | --- |
|  |  | **OTUs** | | | | | | **Sequences** | | | | | |
|  |  | **Non-phasing** | | **One-step phasing** | | **Two-step phasing** | | **Non-phasing** | | **One-step phasing** | | **Two-step phasing** | |
|  |  | **Number** | **% ^c^** | **Number** | **% ^c^** | **Number** | **% ^c^** | **Number** | **% ^c^** | **Number** | **% ^c^** | **Number** | **% ^c^** |
| **Bm1** | **Total OTU** | 36±1 |  | 38±2 |  | 55±5 |  | 21116±3352 |  | 8566±2437 |  | 16356±6088 |  |
|  | **Coverage of the 33 strains** | 31±0 |  | 31±0 |  | 32±0 |  | 21078±3347 |  | 8542±2432 |  | 15386±5742 |  |
|  | **Missing strain(s)** | 2±0 |  | 2±0 |  | 1±0 |  | 9±0 |  | 9±0 |  | 2±3 |  |
|  | **Total Artifacts ^b^** | 5±1 |  | 7±2 |  | 23±5 |  | 38±8 |  | 24±8 |  | 78±31 |  |
|  | **Chimeras ^c^** | 2±1 | 31 | 2±1 | 31 | 1±0 | 3 | 6±3 | 16 | 8±5 | 33 | 4±4 | 5 |
|  | **Contaminants** | 2±1 | 29 | 3±2 | 40 | 8±3 | 34 | 2±1 | 4 | 3±2 | 13 | 12±5 | 15 |
|  | **Erroneous OTUs** | 2±0 | 40 | 2±0 | 29 | 14±3 | 63 | 30±7 | 79 | 13±5 | 54 | 63±27 | 80 |
| **Bm2** | **Total OTU** | 32±2 |  | 33±5 |  | 52±7 |  | 23103±5155 |  | 10886±3702 |  | 18602±8303 |  |
|  | **Coverage of the 33 strains** | 28±2 |  | 27±2 |  | 29±2 |  | 23092±5152 |  | 10876±3698 |  | 18547±8281 |  |
|  | **Missing strain(s)** | 5±2 |  | 6±2 |  | 4±2 |  | 32±13 |  | 42±17 |  | 29±14 |  |
|  | **Total artifacts ^b^** | 4±1 |  | 6±3 |  | 23±6 |  | 11±5 |  | 10±7 |  | 55±26 |  |
|  | **Chimeras ^c^** | 0±0 | 0 | 0±1 | 0 | 0±0 | 0 | 0±0 | 0 | 0±0 | 0 | 0±0 | 0 |
|  | **Contaminants** | 1±1 | 33 | 4±2 | 62 | 8±3 | 35 | 2±2 | 14 | 5±4 | 53 | 12±6 | 21 |
|  | **Erroneous OTUs** | 2±0 | 67 | 2±1 | 38 | 15±3 | 65 | 9±4 | 86 | 5±3 | 47 | 43±20 | 79 |
| **Bm3** | **Total OTU** | 33±3 |  | 30±3 |  | 53±5 |  | 20327±3600 |  | 8309±1670 |  | 16893±5323 |  |
|  | **Coverage of the 33 strains** | 27±2 |  | 24±1 |  | 29±1 |  | 20293±3595 |  | 8281±1664 |  | 16819±5299 |  |
|  | **Missing strain(s)** | 6±2 |  | 9±1 |  | 4±1 |  | 9±0 |  | 9±0 |  | 1±0 |  |
|  | **Total artifacts ^b^** | 6±2 |  | 6±2 |  | 24±4 |  | 35±8 |  | 28±11 |  | 74±25 |  |
|  | **Chimeras ^c^** | 2±0 | 28 | 2±1 | 36 | 1±0 | 4 | 23±7 | 68 | 22±10 | 80 | 17±11 | 23 |
|  | **Contaminants** | 1±1 | 17 | 2±1 | 38 | 9±3 | 35 | 1±1 | 3 | 2±2 | 8 | 12±5 | 17 |
|  | **Erroneous OTU** | 3±2 | 55 | 2±1 | 26 | 15±2 | 61 | 10±4 | 29 | 3±2 | 12 | 44±14 | 60 |

^a^ All data is presented as mean ± s.e. calculated from 24 replicates for each method.

^b^ Total artifacts in the detected OTUs, including chimera, contaminants, and erroneous OTUs. Chimeric sequences were identified based on predictions by UCHIME2 algorithm from USEARCH using the mock community strains as a reference. FASTA sequences for all artifacts were used as query for BLAST search against nt database from NCBI and the mock community strains as reference database. For non-chimera artifacts, all sequences were matched to the nt database from NCBI with a minimum identity of 85%. Those not matched to the mock community strains by BLAST are defined as contaminants, while those matched to the mock community strains by BLAST with the identity above 70% are defined as erroneous OTUs.

^c^ Percentages of chimera, contaminants, and erroneous OTUs in the artifacts.

| **Table S5.** **Sources of artifacts when zOTUs were detected and classed by UPARSE and a sensitive mode was used for chimera identification ^a^** | | | | | | | | | | | | | |
| --- | --- | --- | --- | --- | --- | --- | --- | --- | --- | --- | --- | --- | --- |
|  |  | **zOTUs** | | | | | | **Sequences** | | | | | |
|  |  | **Non-phasing** | | **One-step phasing** | | **Two-step phasing** | | **Non-phasing** | | **One-step phasing** | | **Two-step phasing** | |
|  |  | **Number of zOTUs** | **% ^c^** | **Number of zOTUs** | **% ^c^** | **Number of zOTUs** | **% ^c^** | **Number of Sequences** | **% ^c^** | **Number of Sequences** | **% ^c^** | **Number of Sequences** | **% ^c^** |
| **Bm1** | **Total artifacts ^b^** | 5±1 |  | 7±2 |  | 23±5 |  | 38±8 |  | 24±8 |  | 78±31 |  |
|  | **Chimera** | 2±1 | 33 | 2±1 | 31 | 5±2 | 21 | 6±4 | 17 | 8±5 | 33 | 16±9 | 20 |
|  | **Contaminants** | 2±1 | 29 | 3±2 | 40 | 7±2 | 31 | 2±1 | 4 | 3±2 | 13 | 11±5 | 13 |
|  | **Erroneous zOTUs** | 2±0 | 38 | 2±0 | 29 | 11±2 | 48 | 30±7 | 79 | 13±5 | 54 | 52±22 | 67 |
| **Bm2** | **Total artefacts^b^** | 4±1 |  | 6±3 |  | 23±6 |  | 11±5 |  | 10±7 |  | 55±26 |  |
|  | **Chimera** | 0±0 | 8 | 0±1 | 6 | 4±2 | 18 | 0±1 | 3 | 1±1 | 5 | 9±5 | 17 |
|  | **Contaminants** | 1±1 | 33 | 4±2 | 62 | 7±3 | 32 | 2±2 | 14 | 5±4 | 53 | 11±6 | 20 |
|  | **Erroneous zOTUs** | 2±0 | 59 | 2±1 | 32 | 11±2 | 50 | 9±4 | 83 | 4±2 | 42 | 35±16 | 63 |
| **Bm3** | **Total artifacts ^b^** | 6±2 |  | 6±2 |  | 24±4 |  | 35±8 |  | 28±11 |  | 74±25 |  |
|  | **Chimera** | 2±1 | 34 | 2±1 | 36 | 5±2 | 21 | 24±7 | 69 | 22±10 | 80 | 26±13 | 35 |
|  | **Contaminants** | 1±1 | 15 | 2±1 | 38 | 8±2 | 31 | 1±1 | 3 | 2±2 | 8 | 11±5 | 15 |
|  | **Erroneous zOTU** | 3±2 | 51 | 2±1 | 26 | 12±2 | 48 | 10±4 | 28 | 3±2 | 12 | 37±13 | 50 |

^a^ All data is presented as mean±s.e. calculated from 24 replicates for each method. The proportion of chimera, contaminants, and erroneous OTUs in artifact sequences are displayed.

^b^ Total artifacts in the detected zOTUs, including Chimera, contamiants, and erroneous zOTUs.

^c^ Percentage of the artifacts.

| **Table S6A.****Methods comparisons in OTU or ASV detection and artifact composition and sources when a balanced mode was used for chimera detection** ^a^ | | | | | | | | |
| --- | --- | --- | --- | --- | --- | --- | --- | --- |
|  |  | **Total zOTU or ASVs** | **Coverage of the 33 strains** | **Missing strain(s)** | **Total artifacts ^b^** | **Chimera** | **Contaminants** | **Erroneous OTUs** |
| **OTUs or ASVs** | **DADA2** | 31±5c | 24±2b | 9±2a | 6±4c | 1±1 (18%) | 1±1 | 4±2 |
|  | **Deblur** | 34±4c | 25±2b | 8±2a | 9±3c | 2±1 | 1±1 | 6±2 |
|  | **UCLUST** | 110±25a | 28±2a | 5±2b | 82±25a | 55±20 | 7±3 | 20±5 |
|  | **UNOISE** | 75±9b | 30±1a | 3±1c | 46±8b | 26±6 | 4±1 | 16±2 |
|  | **UPARSE** | 53±5bc | 29±1a | 4±1bc | 24±4bc | 1±0 | 9±3 | 15±2 |
| **Sequences** | **DADA2** | 16148±4970a | 16027±4865a | 9±0a | 121±190a | 9±15 | 3±4 | 109±176 |
|  | **Deblur** | 13555±4141ab | 13518±4123ab | 6±4b | 37±20a | 16±13 | 2±3 | 19±9 |
|  | **UCLUST** | 9460±2976b | 9261±2896b | 0±0c | 199±97a | 147±82 | 9±5 | 43±20 |
|  | **UNOISE** | 16933±5346a | 16740±5263a | 0±0c | 193±96a | 136±80 | 8±4 | 48±18 |
|  | **UPARSE** | 16893±5323a | 16819±5299a | 1±0c | 74±25a | 17±11 | 12±5 | 44±14 |

^a^ All data is presented as mean±s.e. calculated from 24 replicates for each method. Significant differences between methods are shown by alphabetic letters using pairwise T-Test (P < 0.05). The proportion of chimera, contaminants, and erroneous OTUs in artifact sequences are displayed.

^b^ Total artifacts in the detected zOTUs, including Chimera, contaminants, and erroneous zOTUs.

| **Table S6B.** **Methods comparisons in OTU or ASV detection and artifact composition and sources when a sensitive mode was used for chimera identification** ^a^ | | | | | | | | |
| --- | --- | --- | --- | --- | --- | --- | --- | --- |
|  |  | Total zOTU or ASVs | Coverage of the 33 strains | Missing strain(s) | Total artifacts b | Chimera | Contaminants | Erroneous OTUs |
| OTUs or ASVs | DADA2 | 31±5c | 24±2b | 9±2a | 6±4c | 2±1 | 1±1 | 4±2 |
|  | Deblur | 34±4c | 25±2b | 8±2a | 9±3c | 3±1 | 0±1 | 5±2 |
|  | UCLUST | 110±25a | 28±2a | 5±2b | 82±25a | 62±21 | 7±3 | 14±3 |
|  | UNOISE | 75±9b | 30±1a | 3±1c | 46±8b | 30±7 | 4±1 | 11±2 |
|  | UPARSE | 53±5bc | 29±1a | 4±1bc | 24±4bc | 5±2 | 8±2 | 12±2 |
| Sequences | DADA2 | 16148±4970a | 16027±4865a | 9±0a | 121±190a | 12±15 | 3±4 | 106±177 |
|  | Deblur | 13555±4141ab | 13518±4123ab | 6±4b | 37±20a | 18±13 | 1±2 | 18±9 |
|  | UCLUST | 9460±2976b | 9261±2896b | 0±0c | 199±97a | 157±83 | 8±4 | 34±18 |
|  | UNOISE | 16933±5346a | 16740±5263a | 0±0c | 193±96a | 151±86 | 8±4 | 34±12 |
|  | UPARSE | 16893±5323a | 16819±5299a | 1±0c | 74±25a | 26±13 | 11±5 | 37±13 |

^a^ All data is presented as mean±s.e. calculated from 24 replicates for each method. Significant differences between methods are shown by alphabetic letters using pairwise T-Test (P < 0.05). The proportion of chimera, contaminants, and erroneous OTUs in artifact sequences are displayed.

^b^ Total artifacts in the detected zOTUs, including Chimera, contamiants, and erroneous zOTUs.

| **Table S7. Contaminants (ASVs or (z)OTUs) detected from Bm3 using different data processing methods** | | | | | |
| --- | --- | --- | --- | --- | --- |
| **NCBI accession # ^a^** | **Detected by** | **Domain** | **Phylum** | **Species** | **Origin^b^** |
| **MK578773** | DADA2, UCLUST, UNOISE, UPARSE | Archaea | *Candidatus Thermoplasmatota* | unclear *Thermoplasmata archaeon* clone YOBP4 | Archaea mock community |
| **MN100310** | DADA2, Deblur, UCLUST, UNOISE, UPARSE | Archaea | *Candidatus Thermoplasmatota* | *Ferroplasma acidiphilum* | Archaea mock community |
| **JQ346779** | UCLUST | Archaea | *Crenarchaeota* | unclear *Sulfolobales archaeon* clone YOBP2 | Archaea mock community |
| **NR_102972** | UCLUST, UPARSE | Archaea | *Crenarchaeota* | *Caldivirga maquilingensis* | Archaea mock community |
| **DQ924708** | UCLUST | Archaea | *Crenarchaeota* | *Caldivirga maquilingensis* | Archaea mock community |
| **KF607848** | UCLUST, UPARSE | Archaea | *Euryarchaeota* | *Methanobrevibacter smithii* | Archaea mock community |
| **LC183839** | UCLUST | Archaea | [*Euryarchaeota*](https://en.wikipedia.org/wiki/Euryarchaeota) | *Methanolacinia paynteri* | Archaea mock community |
| **MN505783** | UCLUST, UPARSE | Archaea | *Euryarchaeota* | *Methanobrevibacter arboriphilus* | Archaea mock community |
| **MG430446** | UCLUST | Archaea | *Euryarchaeota* | unclassified | Archaea mock community |
| **KX133611** | UCLUST | Archaea | *Euryarchaeota* | *Thermococcus guaymasensis* | Archaea mock community |
| **KR136084** | UCLUST, UPARSE | Archaea | *Euryarchaeota* | *Methanothermobacter thermoautotrophicus* | Archaea mock community |
| **MK680235** | UCLUST, UPARSE | Archaea | *Euryarchaeota* | *Methanobacterium bryantii* | Archaea mock community |
| **CP020120** | UCLUST, UPARSE | Archaea | *Euryarchaeota* | *Methanococcus maripaludis* | Archaea mock community |
| **KT068037** | UCLUST, UNOISE, UPARSE | Archaea | *Euryarchaeota* | *Halomicrobium katesii* | Archaea mock community |
| **NR_118366** | UCLUST, UPARSE | Archaea | *Euryarchaeota* | *Methanospirillum hungatei* | Archaea mock community |
| **MH205989** | UCLUST, UPARSE | Archaea | *Euryarchaeota* | *Methanohalophilus halophilus* | Archaea mock community |
| **KY932730** | UCLUST, UPARSE | Archaea | *Euryarchaeota* | *Methanosphaera stadtmanae* | Archaea mock community |
| **KU030162** | UCLUST, UPARSE | Archaea | *Euryarchaeota* | *Archaeoglobus profundus* | Archaea mock community |
| **MG854222** | DADA2, Deblur, UCLUST, UNOISE, UPARSE | Archaea | *Euryarchaeota* | *Thermoplasma* | Archaea mock community |
| **MW549204** | DADA2, UNOISE, UPARSE | Archaea | *Nitrososphaerota* | *Nitrosopumilus maritimus* | Archaea mock community |
| **KJ881840** | UCLUST | Archaea | *Nitrososphaerota* | *Nitrosopumilus maritimus* | Archaea mock community |
| **MK868065** | UCLUST | Archaea | *Nitrososphaerota* | *Nitrosopumilus maritimus* | Archaea mock community |
| **HM150163** | UCLUST, UNOISE, UPARSE | Archaea | *Thermoproteota* | unclear *Thermoproteales archaeon* clone YOBP5 | Archaea mock community |
| **KT068192** | DADA2, UCLUST, UNOISE, UPARSE | Archaea | *Thermoproteota* | *Thermoprotei* | Archaea mock community |
| **MN024249** | UCLUST, UPARSE | Archaea | [*Thermoproteota*](https://en.wikipedia.org/wiki/Thermoproteota) | *Sulfolobus acidocaldarius* | Archaea mock community |
| **AB681496** | UCLUST | Bacteria | *Bacteroidetes* | *Flavobacterium* | unclassified Flavobacterium |
| **CP050461** | UCLUST | Bacteria | *Bacteroidetes* | *Nonlabens* | unclear |
| **KY275920** | UCLUST | Bacteria | Unclassified | Unclassified | environmental samples |
| **KU506153** | UCLUST | Bacteria | Unclassified | Unclassified | environmental samples |
| **KP944828** | UCLUST, UPARSE | Bacteria | Unclassified | Unclassified | environmental samples |
| **KY278883** | UCLUST | Bacteria | Unclassified | Unclassified | environmental samples |
| **KY276160** | UCLUST, UPARSE | Bacteria | Unclassified | Unclassified | environmental samples |
| **HM839502** | UCLUST | Bacteria | Unclassified | Unclassified | environmental samples |
| **KY278704** | UCLUST | Bacteria | Unclassified | Unclassified | environmental samples |
| **LR639402** | UCLUST | Bacteria | Unclassified | Unclassified | environmental samples |
| **MN891656** | UCLUST | Bacteria | Unclassified | Unclassified | environmental samples |
| **LN516743** | UCLUST | Bacteria | Unclassified | Unclassified | environmental samples |
| **MH315414** | UCLUST | Bacteria | Unclassified | Unclassified | environmental samples |
| **KY275641** | UCLUST | Bacteria | Unclassified | Unclassified | environmental samples |
| **MW082982** | UCLUST | Bacteria | Unclassified | Unclassified | environmental samples |
| **MZ974455** | UCLUST | Bacteria | Unclassified | Unclassified | environmental samples |
| **LC496407** | UCLUST, UPARSE | Bacteria | Unclassified | Unclassified | environmental samples |
| **MF498141** | UCLUST | Bacteria | Unclassified | Unclassified | environmental samples |
| **MG867092** | UCLUST | Bacteria | Unclassified | Unclassified | environmental samples |
| **LR638981** | UCLUST | Bacteria | Unclassified | Unclassified | environmental samples |
| **HQ189625** | UPARSE | Bacteria | Unclassified | Unclassified | environmental samples |
| **OK071313** | UPARSE | Bacteria | Unclassified | Unclassified | environmental samples |
| **LR640988** | UPARSE | Bacteria | Unclassified | Unclassified | environmental samples |
| **MH761168** | Deblur | Bacteria | Unclassified | Unclassified | environmental samples |
| **MN857808** | UCLUST, UPARSE | Bacteria | Unclassified | Unclassified | environmental samples |
| **MF660344** | UCLUST | Bacteria | Unclassified | Unclassified | environmental samples |
| **MH526910** | UCLUST | Bacteria | Unclassified | Unclassified | environmental samples |
| **MF950503** | UCLUST | Bacteria | Unclassified | Unclassified | environmental samples |
| **MH934243** | UPARSE | Bacteria | Unclassified | Unclassified | environmental samples |
| **LC667825** | UCLUST | Bacteria | *Firmicutes* | *Rossellomorea* | unclear |
| **HQ183839** | UCLUST | Bacteria | *Proteobacteria* | Unclassified | environmental samples |
| **OL897515** | UCLUST | Bacteria | *Proteobacteria* | *Diaphorobacter* | unclassified Diaphorobacter |
| **HG986835** | UCLUST | Bacteria | *Proteobacteria* | *Massilia group* | environmental samples |
| **MK603690** | UCLUST, UPARSE | Bacteria | *Proteobacteria* | Unclassified | unclear |
| **MN664230** | DADA2, UCLUST, UNOISE, UPARSE | Bacteria | *Proteobacteria* | *Thiobacillus* | environmental samples |
| **CP053837** | UCLUST | Bacteria | *Proteobacteria* | *Aliarcobacter* | unclear |
| **KC433404** | UCLUST, UPARSE | Bacteria | *Proteobacteria* | *Catenovulum* | unclassified Catenovulum |
| **MZ734445** | UCLUST | Bacteria | *Proteobacteria* | *Providencia* | unclassified Providencia |
| **EF092210** | UCLUST | Bacteria | *Proteobacteria* | *Legionella* | environmental samples |
| **OK464425** | UCLUST | Bacteria | *Proteobacteria* | *Acinetobacter* | unclear |
| **OL630582** | UCLUST, UPARSE | Bacteria | *Proteobacteria* | *Psychrobacter* | unclear |
| **MW287983** | UCLUST | Bacteria | *Proteobacteria* | Unclassified | unclear |
| **KU721136** | UCLUST | Bacteria | *Proteobacteria* | *Methylophaga* | environmental samples |
| **KT039578** | UCLUST | Bacteria | Unclassified | Unclassified | environmental samples |

^a^ All NCBI sequences shown here are matched to detected ASVs or (z)OTUs with identity above 94%.

^b^ Contaminant sequences were used as query for BLAST search against the archaea mock community strains as reference database. The identities between archaea mock community and all matched contaminant sequences are above 98% except for MK868065 (78%).

| **Table S8A.** **Sources of the spurious sequences-Bm1** | | | | | | | |
| --- | --- | --- | --- | --- | --- | --- | --- |
|  | | Non-Phasing | | One-step phasing | | Two-step phasing | |
|  |  | number | % | number | % | number | % |
| Total Spurious |  | 516 |  | 495 |  | 465 |  |
| Singletons | total | 504 | 97.7 | 487 | 98.4 | 457 | 98.3 |
|  | chimera | 376 | 74.6 | 358 | 73.5 | 221 | 48.4 |
|  | True Positive | 0 | 0.0 | 0 | 0.0 | 0 | 0.0 |
|  | E. coli | 4 | 0.8 | 0 | 0.0 | 0 | 0.0 |
|  | other contaminant strains | 44 | 8.7 | 44 | 9.0 | 80 | 17.5 |
|  | erroneous sequences | 80 | 15.9 | 85 | 17.5 | 156 | 34.1 |
| Doubletons | total | 12 | 2.3 | 8 | 1.6 | 8 | 1.7 |
|  | chimera | 9 | 75.0 | 6 | 75.0 | 6 | 75.0 |
|  | True Positive | 0 | 0.0 | 0 | 0.0 | 0 | 0.0 |
|  | E. coli | 0 | 0.0 | 0 | 0.0 | 0 | 0.0 |
|  | other contaminant strains | 1 | 8.3 | 2 | 25.0 | 1 | 12.5 |
|  | erroneous sequences | 2 | 16.7 | 0 | 0.0 | 1 | 12.5 |
| Other Unique OTUs ^a^ | total | 0 | 0.0 | 0 | 0.0 | 0 | 0.0 |
|  | chimera | 0 | 0.0 | 0 | 0.0 | 0 | 0.0 |
|  | True Positive | 0 | 0.0 | 0 | 0.0 | 0 | 0.0 |
|  | E. coli | 0 | 0.0 | 0 | 0.0 | 0 | 0.0 |
|  | other contaminant strains | 0 | 0.0 | 0 | 0.0 | 0 | 0.0 |
|  | erroneous sequences | 0 | 0.0 | 0 | 0.0 | 0 | 0.0 |

^a^ OTUs have ≥ 3 sequences and present in only one library across the entire experiments.

| **Table S8B.** **Sources of the spurious sequences-Bm2 ^a^** | | | | | | | |
| --- | --- | --- | --- | --- | --- | --- | --- |
|  | | None-Phasing | | One-step phasing | | Two-step phasing | |
|  |  | number | % | number | % | number | % |
| Total Spurious |  | 183 |  | 202. |  | 284 |  |
| Singletons | total | 182 | 99.5% | 200 | 99.0% | 278 | 97.9% |
|  | chimera | 78 | 42.9% | 85 | 42.5% | 71 | 25.5% |
|  | True Positive | 0 | 0.0% | 0 | 0.0% | 0 | 0.0% |
|  | E. coli | 2 | 1.1% | 0 | 0.0% | 0 | 0.0% |
|  | other contaminant strains | 29 | 15.9% | 31 | 15.5% | 69 | 24.8% |
|  | erroneous sequences | 73 | 40.1% | 84 | 42.0% | 138 | 49.6% |
| Doubletons | total | 1 | 0.6% | 2 | 1.0% | 5 | 1.8% |
|  | chimera | 0 | 0.0% | 1 | 50.0% | 1 | 20.0% |
|  | True Positive | 0 | 0.0% | 0 | 0.0% | 0 | 0.0% |
|  | E. coli | 0 | 0.0% | 0 | 0.0% | 0 | 0.0% |
|  | other contaminant strains | 1 | 100.0% | 1 | 50.0% | 1 | 20.0% |
|  | erroneous sequences | 0 | 0.0% | 0 | 0.0% | 3 | 60.0% |
| Other Unique OTUs | total | 0 | 0.0% | 0 | 0.0% | 1 | 0.4% |
|  | chimera | 0 | 0.0% | 0 | 0.0% | 0 | 0.0% |
|  | True Positive | 0 | 0.0% | 0 | 0.0% | 0 | 0.0% |
|  | E. coli | 0 | 0.0% | 0 | 0.0% | 0 | 0.0% |
|  | other contaminant strains | 0 | 0.0% | 0 | 0.0% | 1 | 0.4% |
|  | erroneous sequences | 0 | 0.0% | 0 | 0.0% | 0 | 0.0% |

| **Table S8C.** **Sources of the spurious sequences-Bm3 ^a^** | | | | | | | |
| --- | --- | --- | --- | --- | --- | --- | --- |
|  | | None-Phasing | | One-step phasing | | Two-step phasing | |
|  |  | number | % | number | % | number | % |
| Total Spurious |  | 298 |  | 278 |  | 280 |  |
| Singletons | total | 295 | 97.7% | 274 | 98.6% | 274 | 97.9% |
|  | chimera | 185 | 62.7% | 185 | 67.5% | 90 | 32.9% |
|  | True Positive | 0 | 0.0% | 2 | 0.73% | 1 | 0.4% |
|  | E. coli | 2 | 0.7% | 0 | 0.0% | 0 | 0.0% |
|  | other contaminant strains | 28 | 9.5% | 37 | 13.5% | 87 | 31.8% |
|  | erroneous sequences | 80 | 27.1% | 50 | 18.3% | 96 | 35.0% |
| Doubletons | total | 3 | 1.0% | 4 | 1.4% | 6 | 2.1% |
|  | chimera | 1 | 33.3% | 4 | 100.0% | 3 | 50.0% |
|  | True Positive | 0 | 0.0% | 0 | 0.0% | 0 | 0.0% |
|  | E. coli | 0 | 0.0% | 0 | 0.0% | 0 | 0.0% |
|  | other contaminant strains | 1 | 33.3% | 0 | 0.0% | 1 | 16.7% |
|  | erroneous sequences | 1 | 33.3% | 0 | 0.0% | 2 | 33.3% |
| Other Unique OTUs | total | 0 | 0.0% | 0 | 0.0% | 0 | 0.0% |
|  | chimera | 0 | 0.0% | 0 | 0.0% | 0 | 0.0% |
|  | True Positive | 0 | 0.0% | 0 | 0.0% | 0 | 0.0% |
|  | E. coli | 0 | 0.0% | 0 | 0.0% | 0 | 0.0% |
|  | other contaminant strains | 0 | 0.0% | 0 | 0.0% | 0 | 0.0% |
|  | erroneous sequences | 0 | 0.0% | 0 | 0.0% | 0 | 0.0% |

| **Table S11. Mock Community strains** | | | | | | | |
| --- | --- | --- | --- | --- | --- | --- | --- |
|  | Strains | Group ^a^ | Abundance (%) | | | V3-V5 GC (%) | V4 GC (%) |
|  |  |  | Bm1 | Bm2 | Bm3 |  |  |
| 1 | Acidobacteria_JQ346769 | Low GC | 3.03 | 8.41 | 0.01 | 53 | 55.82 |
| 2 | Bacteroidetes_clone1_JQ346767 | Low GC | 3.03 | 8.41 | 0.01 | 50 | 47.26 |
| 3 | Syntrophobacter_fumaroxidans_JQ346744 | Low GC | 3.03 | 8.41 | 0.01 | 57 | 56.32 |
| 4 | Protochlamydia_amoebophila_JQ346728 | Low GC | 3.03 | 8.41 | 0.01 | 51 | 51.37 |
| 5 | Chlorobi_JQ346768 | Low GC | 3.03 | 8.41 | 0.01 | 52 | 53.42 |
| 6 | Desulfurispirillum_alkaliphilum_JQ346730 | Low GC | 3.03 | 8.41 | 0.01 | 53 | 50.68 |
| 7 | Cyanobacterium_JQ346766 | Low GC | 3.03 | 8.41 | 0.01 | 47 | 45.55 |
| 8 | Syntrophococcus_sucromutans_JQ346731 | Low GC | 3.03 | 8.41 | 0.01 | 53 | 54.11 |
| 9 | Leptotrichia_hofstadii_JQ346732 | Low GC | 3.03 | 8.41 | 0.01 | 53 | 54.11 |
| 10 | Victivallis_vadensis_JQ346729 | Low GC | 3.03 | 8.41 | 0.01 | 52 | 50 |
| 11 | Mycoplasma_orale_JQ346727 | Low GC | 3.03 | 8.41 | 0.01 | 49 | 49.83 |
| 12 | Actinobacterium_JQ346771 | Medium GC | 3.03 | 0.67 | 0.67 | 49 | 53.42 |
| 13 | Persephonella_hydrogeniphila_JQ346733 | Medium GC | 3.03 | 0.67 | 0.67 | 60 | 59.25 |
| 14 | Caldisericum_exile_JQ346734 | Medium GC | 3.03 | 0.67 | 0.67 | 55 | 54.11 |
| 15 | Deinococcus_indicus_JQ346735 | Medium GC | 3.03 | 0.67 | 0.67 | 55 | 56.51 |
| 16 | Desulfovibrio_AJ786059 | Medium GC | 3.03 | 0.67 | 0.67 | 54 | 55.82 |
| 17 | Planctomycete_JQ346772 | Medium GC | 3.03 | 0.67 | 0.67 | 55 | 54.79 |
| 18 | Syntrophus_buswellii_JQ346736 | Medium GC | 3.03 | 0.67 | 0.67 | 54 | 54.11 |
| 19 | Syntrophus_gentianae_JQ346737 | Medium GC | 3.03 | 0.67 | 0.67 | 53 | 54.45 |
| 20 | Spirochaetes_JQ346773 | Medium GC | 3.03 | 0.67 | 0.67 | 55 | 54.45 |
| 21 | Synergistetes_JQ346774 | Medium GC | 3.03 | 0.67 | 0.67 | 55 | 54.11 |
| 22 | Verrucomicrobia_JQ346775 | Medium GC | 3.03 | 0.67 | 0.67 | 55 | 55.14 |
| 23 | Sulfurihydrogenibium_yellowstonense_JQ346738 | High GC | 3.03 | 0.01 | 8.41 | 56 | 56.51 |
| 24 | Thermomicrobium_roseum_JQ346739 | High GC | 3.03 | 0.01 | 8.41 | 67 | 68.84 |
| 25 | Deferribacter_desulfuricans_JQ346740 | High GC | 3.03 | 0.01 | 8.41 | 59 | 59.25 |
| 26 | Dictyoglomus_thermophilum_JQ346741 | High GC | 3.03 | 0.01 | 8.41 | 59 | 59.59 |
| 27 | Fibrobacter_succinogenes_JQ346742 | High GC | 3.03 | 0.01 | 8.41 | 56 | 57.19 |
| 28 | Syntrophothermus_lipocalidus_JQ346743 | High GC | 3.03 | 0.01 | 8.41 | 57 | 57.88 |
| 29 | Gemmatimonadetes_JQ346776 | High GC | 3.03 | 0.01 | 8.41 | 61 | 62.33 |
| 30 | Nitrospira_JQ346777 | High GC | 3.03 | 0.01 | 8.41 | 57 | 59.93 |
| 31 | Bacteroidetes_clone2_JQ346770 | High GC | 3.03 | 0.01 | 8.41 | 55 | 50 |
| 32 | Thermodesulfobacterium_commune_JQ346745 | High GC | 3.03 | 0.01 | 8.41 | 61 | 60.96 |
| 33 | Thermotoga_neapolitana_JQ346746 | High GC | 3.03 | 0.01 | 8.41 | 62 | 63.7 |

^a^ Average GC content: Low GC group, 51.1±2.1%; medium GC group: 55.1±1.8%; high GC group, 59.3±3.3%.
